# Mammalian/mechanistic target of rapamycin (mTOR) complexes in neurodegeneration

**DOI:** 10.1186/s13024-021-00428-5

**Published:** 2021-07-02

**Authors:** Henry Querfurth, Han-Kyu Lee

**Affiliations:** Department of Neurology, Tufts Medical Center, Boston, Massachusetts USA

**Keywords:** Alzheimer’s, mTOR, Rapamycin, Insulin signaling, Akt, Parkinson’s

## Abstract

Novel targets to arrest neurodegeneration in several dementing conditions involving misfolded protein accumulations may be found in the diverse signaling pathways of the Mammalian/mechanistic target of rapamycin (mTOR). As a nutrient sensor, mTOR has important homeostatic functions to regulate energy metabolism and support neuronal growth and plasticity. However, in Alzheimer’s disease (AD), mTOR alternately plays important pathogenic roles by inhibiting both insulin signaling and autophagic removal of β-amyloid (Aβ) and phospho-tau (ptau) aggregates. It also plays a role in the cerebrovascular dysfunction of AD. mTOR is a serine/threonine kinase residing at the core in either of two multiprotein complexes termed mTORC1 and mTORC2. Recent data suggest that their balanced actions also have implications for Parkinson's disease (PD) and Huntington's disease (HD), Frontotemporal dementia (FTD) and Amyotrophic Lateral Sclerosis (ALS). Beyond rapamycin; an mTOR inhibitor, there are rapalogs having greater tolerability and micro delivery modes, that hold promise in arresting these age dependent conditions.

## Introduction

Aging represents the major biologic process common to most all neurodegenerations, driving the accumulation of damaging changes to organ systems and their cells over time. The core metabolic pathologies involved in chronic disease states of the central nervous system (CNS) are oxidative stress, inflammation, mitochondrial/energy failure and insulin resistance [1–3]. Neurodegenerative disorders are further distinguished from other chronic disease conditions such as cancer and cardiovascular disease by the deposition of characteristic misfolded proteins. Nevertheless, certain essential, universally shared cell signaling pathways become deranged in all of them. Mechanistic target of rapamycin (mTOR) refers to two protein complexes, mTORC1 and mTORC2, that function as master switches in the cell's nutrient sensing pathways. The mTOR signaling pathway integrates extracellular growth factors and cellular nutrient status to regulate growth and metabolism during aging [4, 5]. It is of major relevance to neurodegeneration that intact mTOR signaling is critical to long lasting forms of synaptic plasticity (NMDA-R-dependent late phase LTP and mGlu-R-dependent LTD) [6, 7], as well as to spatial learning [8]. The evidence points in support of de novo synaptic protein synthesis by mTOR [9, 10]. Further, mTOR is necessary for dendritic spine morphological changes in association with LTP induction [11].

On the other hand, there is overwhelming evidence that decreasing mTORC1 activity, for instance via caloric restriction [12] or through dietary administration of rapamycin , increases lifespan in model organisms, including yeast, *C. elegans*, *D. melanogaster,* and mice [13, 14]. Even mice fed rapamycin beginning in later life lived longer [13–15]. It is noteworthy that primates also had extended lifespans with fewer age-related pathologies when calorically restricted beginning in adulthood. This includes preservation of brain grey matter volume [16]. In addition to the above physiologic roles, the central role of mTORC1 signaling in the development of neurodegenerative diseases is a topic of great research and therapeutic interest. This is, in part, because mTORC1 not only supports protein synthesis via translation regulation, but controls both protein and organelle degradation through autophagy. The autophagy / lysosomal system is a cellular recycling process required to prevent the buildup of misfolded protein aggregates that contribute to the development of neurodegenerative diseases. These include β-amyloid (Aβ) and phospho-tau (p-tau) oligomers in Alzheimer's disease (AD) and α-synuclein in Parkinson’s disease (PD), discussed below.

Herein, we first review the mTOR pathways and their regulation, prior to discussing complex changes in mTOR activity as are reported in various AD models. This is followed by summarizing its contribution to the pathogenesis of lesser studied neurodegenerative diseases including Parkinson’s disease (PD), Huntington's disease (HD), Amyotrophic Lateral Sclerosis (ALS), and Frontotemporal dementia (FTD). Throughout we emphasize alterations in autophagy and insulin signaling. The rationale and prospects for the treatment of neurodegeneration across disease contexts is laid out, based on both beneficial and deleterious effects of mTORC1 inhibition. We conclude with an argument favoring a balance involving mTORC2 stimulation.

## Mechanistic Target of Rapamycin: Pathways and Regulation

### mTORC1

The mTOR complex 1 (mTORC1) is comprised of the 289 kDa mTOR serine-threonine kinase, its rapamycin-sensitive regulatory protein Raptor, as well as three other proteins: GβL/mLST8, a 40 kDa proline-rich Akt substrate (PRAS40) and Deptor. It acts as a crucial cellular energy and nutrient sensor as well as growth factor (Insulin, IGF-1, BDNF) transducer. mTORC1 controls protein synthesis by phosphorylating downstream targets essential to mRNA translation, 4E-BP1 (eIF-4E binding protein) and ribosome biogenesis, p70S6K1 (p70 ribosomal protein S6 kinase 1) [17]. As synaptic plasticity and dendritic spine maintenance require *de novo* protein synthesis, mTORC1 supports the biological processes that underlie learning and long-term memory [17–19]. Accordingly, neuronal growth factors known to support learning and memory, such as BDNF and EGF, do so through mTOR activation [20, 21]. By contrast, the pharmacologic mTORC1 inhibitor, rapamycin, or genetic reductions in mTORC1, block several types of memory consolidation such as fear conditioning and late phase-LTP (long-term potentiation) [6, 7, 22]. Analogous to brain, knockout of either Raptor or mTOR or application of specific inhibitors in skeletal muscle results in a muscular dystrophy [23, 24]. Inhibition of autophagy (see below) and stimulation of mitochondrial respiration are other key mTOR roles [25, 26] affecting cell growth, division, proliferation, survival and aging.

**Fig. 1:**
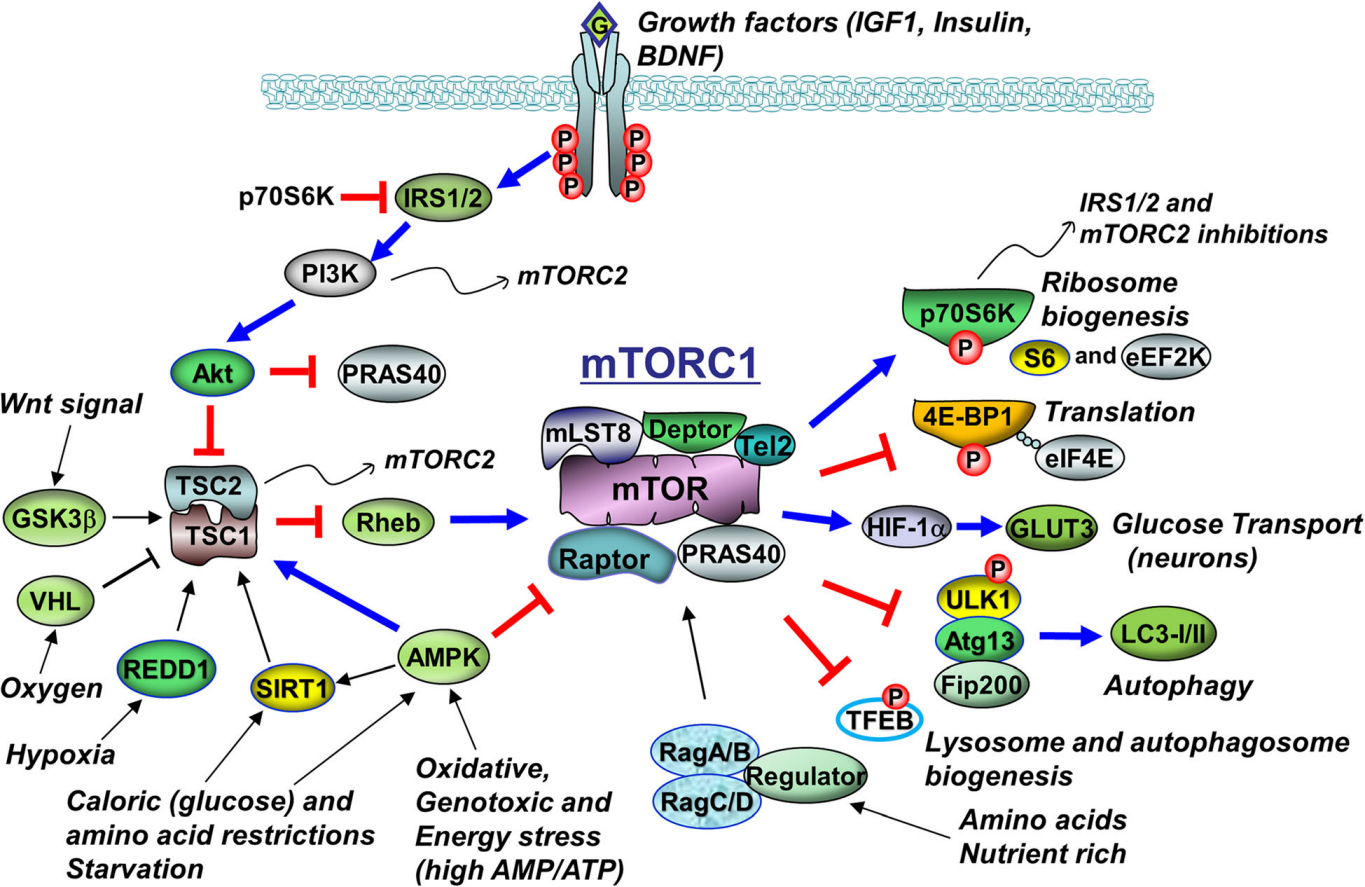
mTORC1 pathways. Growth factor/neurotrophin, energy, nutrient and oxygen tension state inputs are shown. Effects on protein synthesis, glucose transport, autophagy and cell growth/survival are indicated. PRAS and Deptor are negative C1 regulatory units. Caloric restriction/AMPK and amino acid restriction through TSC1/2 are important C1-inhibitory paths. Negative feedback onto insulin/PI3K/Akt from C1 is noted

The Tuberous Sclerosis protein complex, TSC1/2, proximally inhibits mTORC1 by preventing the conversion of the mTORC1 activator, Rheb (Ras homolog enriched in brain protein), into its active GTP-bound form (Fig. 1). Insulin/Akt signaling leads to the inactivating phosphorylation of TSC1/2, thus, activated Akt can release mTOR from TSC1/2-mediated inhibition [27, 28]. Neurotrophin-induced activation of mTOR takes place at lysosomal and plasma membranes [29, 30].

AMP-activated protein kinase (AMPK), another important nutrient sensor and cell energy broker that is activated by low substrate (glucose) levels and low ATP conditions (high ADP-AMP/ATP ratio), i.e. energy stress, is an important negative regulator of mTOR [31, 32]. Whereas insulin/Akt signaling leads to the inhibitory phosphorylation of TSC2 (on Ser-939, Ser-1088, and Thr-1422), AMPK phosphorylates TSC2 on a stimulatory residue (Ser-1387) [33–35]. AMPK itself is also negatively regulated by Akt [36]. By inhibiting mTORC1 via TSC2, one consequence of activated AMPK is to facilitate autophagy [37, 38]. Another is to inhibit protein translation [31]. A second mechanism by which AMPK inhibits protein synthesis is by phospho-activating the elongation factor kinase, eEF2K (pS398). The target of eEF2K action is eEF2, which becomes inhibited (pT56). The consequence is to turn off the elongation step in mRNA translation [39, 40]. This action of AMPK therefore opposes the effect of mTORC1/p70S6K, which is to inhibit eEF2K (pS366). On the other hand when AMPK is inhibited, for instance in the palmitate model of insulin resistance, mTOR is stimulated [41]. Under the latter condition, as well under others such as endoplasmic reticulum stress and apoptosis, mTOR induction becomes detrimental to cell health. Rapamycin reverses this process by binding to FK506-binding protein- 12 (FKBP12) in a complex that allosterically blocks the catalytic activity of the mTOR subunit [41–46]. Akt and AMPK can also bypass TSC, to oppositely influence mTOR directly, via PRAS40 and Raptor phosphorylations, respectively.

One target of mTORC1, p70S6K, has an additional negative feedback role to down-regulate insulin/Akt signaling through an inactivating phosphorylation of the insulin receptor substrate 1 (IRS-1) [47] (Fig. 1). This function of stimulated mTORC1 to negatively regulate sustained Akt activation by insulin [48, 49] has central importance to the widely held notion that the AD brain is an insulin resistant organ [50, 51].

In synergy with insulin, the branched chain amino acids (BCAAs), especially leucine, potently stimulate mTOR to induce protein synthesis (reviewed in [52, 53]). It has long been known that mTOR is a nutrient sensor for amino acids in an Akt-independent manner [54, 55]. Insight in this field came from studies on rodent and human skeletal muscle where infusion or ingestion of leucine (often in combination with resistance exercise) was tested to combat age-related muscle mass (sarcopenia) [56–58]. Mechanistically, leucine administration causes the mTORC1-mediated phosphorylation of both 4E-BP1 and p70S6K1, two proteins that each play critical roles in mRNA translation (4E-BP1 facilitates the interaction between the 5’-cap and the 40S ribosome, whereas p70S6K enables translation of polypyrimidine mRNAs) [59, 60]. As a result, BCAAs in normal concentrations induce protein synthesis in an mTORC1-dependent manner.

More recently, the mechanism of BCAA-induced mTOR stimulation has been expanded to involve a multiprotein complex on the lysosomal surface composed of: Rags (Ras-related GTPases), Ragulator (an anchoring protein) and the vacuolar (H+)-ATPase (responsible for endosomal and lysosomal acidification) [61–63]. Another complex with similar function consists of hVps34 (human vacuolar sorting protein 34) and phospholipase D1. These complexes recruit mTOR to the lysosomal surface, where it is activated by Rheb. Since mTOR is activated at the lysosome membrane and amino acids enable its translocation to the lysosome, normal mTOR activity is dependent on these anabolic amino acids [64].

The contribution of amino acid availability to mTORC1 activity in neurodegenerative disease is unknown. However, it’s worth noting that BCAA levels are positively associated with obesity and to the development of type II diabetes mellitus (T2DM) [65, 66]. Further evidence suggests that excess BCAA may be a marker of insulin resistance or even be causative [67, 68]. Considering that insulin resistance is an underlying pathology of several neurodegenerative diseases, it is plausible that excess BCAAs persistently overstimulate mTORC1, resulting in the p70S6K1-mediated negative feedback on IRS-1. The consequential uncoupling of insulin action, in turn, could promote protein catabolism and an increase in harmful aminoacidemia. Furthermore, the metabolites of BCAAs are mitochondrial toxins, thus inducing oxidative stress and further exacerbating the CNS insulin resistant phenotype. On the one hand, amino acid depletion is catabolic and stimulates autophagy. Also, as noted, supplemental BCAA might be beneficial in some instances. The negative role of excess BCAAs in neurodegenerative disease is speculative, but deserves further exploration.

Lastly, SIRT1, an NAD+-dependent and resveratrol-responsive deacetylase that enables caloric restriction-mediated longevity, negatively regulates mTORC1 [69] by interacting with TSC1/2 [70].

### mTORC2

mTORC2 is a relatively (though not completely) rapamycin-resistant complex comprising mTOR and Rictor (rapamycin-insensitive companion of mTOR; thereby contrasting Raptor in mTORC1), in addition to GβL/mLST8, mSIN1, PRR5/Protor and Deptor proteins. Therefore, both mTOR complexes share mTOR, mLST8 and Deptor, and are distinguished by Raptor and PRAS40 (mTORC1) and Rictor, mSIN1, and Protor (mTORC2).

**Fig. 2:**
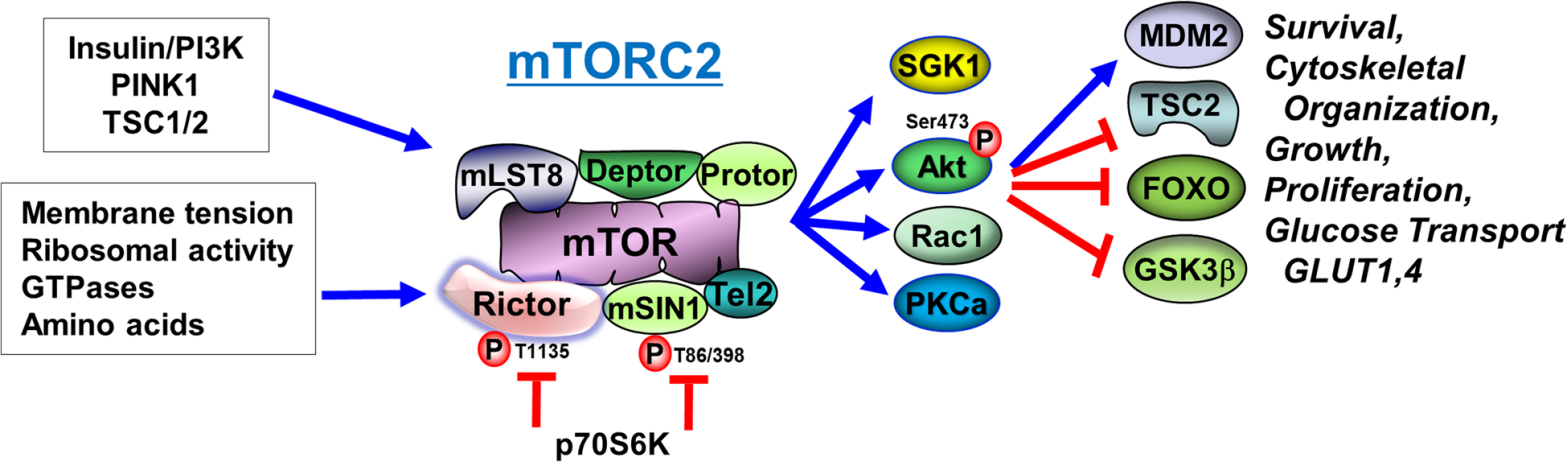
mTORC2 pathways. The regulation of C2 is less clear, but is also responsive to the neurotrophins. Positive feedback onto Akt from C2 is noted. See abbreviations

mTORC2 also differs from mTORC1 in terms of its regulation. mTORC1 and 2 are inhibited and activated, respectively, by TSC1/2 [34, 71]. Moreover, while not directly regulated by nutrients, mTORC2 is activated by trophic factors insulin/IGF-1 in a yet to be defined manner that requires PI3K and involves ribosome binding [72] (Fig. 2).

In terms of downstream targets, mTORC2 amplifies the activation of Akt by acting as an Akt-kinase (PDK2) (see excellent reviews: [73, 74]). mTORC2 phosphorylates Akt on Ser-473, secondarily to the phosphorylation of Thr-308 by PDK1 [75–81]. Ser-473 phosphorylation is required for full Akt activation. When mTORC2 function is impaired in skeletal muscle for instance, Akt function is diminished and glucose intolerance ensues [82]. Thus, mTORC2 opposes the indirect negative regulation of Akt by the mTORC1/IRS-1 pathway. Other targets of mTORC2 include the actin cytoskeleton and serum/glucocorticoid-regulated kinase 1 (SGK-1), which activates certain ion channels and regulates cell volume and growth [83, 84]. Thus, mTORC2 is important in cytoskeleton remodeling and electrolyte homeostasis. In the mouse CNS, conditional ablation adversely affects neuron morphology and post-synaptic excitatory currents [85], attesting to its functional importance in the brain.

Consistent with both mTOR complexes as have opposing effects on Akt, it makes sense that mTORC1 can also inhibit mTORC2 via Rictor (Thr-1135) and mSin1 phosphorylations through p70S6k1 action, in order to further dampen insulin/Akt signaling as part of a negative feedback system [86, 87]. Consequently, selective inhibition of mTORC1 with short-term rapamycin treatment (or with mTORC1-specific compounds [88]) may activate Akt by both relieving p70S6K-mediated IRS-1/Akt suppression [47] and permitting mTORC2-mediated activation. Long-term rapamycin on the other hand disassembles mTORC2, ultimately leading to insulin resistance [78, 89–91]. It is interesting that mTORC2 can mediate the degradation of IRS-1 that has been inactivated (pS307) by persistent mTORC1 [92].

## mTOR control of protein synthesis and autophagy

mTOR’s control of protein synthesis and autophagy are thought to account for its contribution to neurodegenerative diseases. In general, mTOR supports protein synthesis by regulating cap-dependent translation through the phosphorylations of p70S6K and 4E-BP1 [72]. Rapamycin and related mTOR antagonists inhibit protein manufacturing in response to the cell's energy needs and dietary state [93]. By supporting protein synthesis in dendrites and their synapses [17], mTOR promotes synaptic plasticity [7, 94] and hippocampal memory consolidation and maintenance [95, 96].

Parkinson’s disease protein 7 (PARK7, a.k.a. DJ1) is an example of a protein relevant to neurodegeneration, whose translation depends on mTORC1. PARK7/DJ1 has beneficial chaperone and anti-oxidant properties. Loss of function mutations in PARK7 cause early onset, recessive PD. Inhibition of mTORC1 with rapamycin reduced neuroprotective PARK7/DJ1 levels in rodent cortical synapses. Conversely, genetic over-activation of mTOR (using a TSC1 knock-out Tuberous Sclerosis disease model) doubles normal PARK7 levels. This observation highlights the nuance of therapeutically targeting mTOR, suggesting that blindly inhibiting mTORC1 could have negative consequences in certain conditions.

Autophagy (here referring to macroautophagy, as opposed to the lesser-studied microautophagy and chaperone-mediated autophagy), is a conserved cellular pathway for removing unnecessary or toxic protein aggregates and recycling damaged organelles (particularly mitochondria). Autophagy involves the formation of a double-membrane autophagosome around the protein or organelle targets, that later fuse with lysosomes, resulting in their degradation. It is activated by low nutrient availability, as well as by protein aggregation and organelle damage [97]. mTORC1 is a master negative regulator of autophagy, as mTORC1-mediated phosphorylations block complex formation between Atg13 (autophagy-related gene product) and the autophagy initiation protein, ULK1/2 (Unc-51 like kinase), thereby preventing autophagosome formation [98, 99]. mTORC1 further suppresses the induction of autophagy by interfering with AMPK's direct phosphorylation of ULK1/2 [100]. Other mechanisms include negatively regulating TFEB, the transcription factor responsible for lysosomal biogenesis and inhibiting the activation of LC3BI/II [101] (Fig. 1). It should be mentioned that autophagy begins to fail in neurodegenerative disorders because itself becomes the target of toxic protein oligomers [97, 102].

The essential role of autophagy in neurodegeneration is demonstrated by the deletion of autophagy genes (Atgs), which results in age-dependent neurodegeneration and proteostasis in model systems [103, 104]. Pharmacological downregulation of mTORC1 enhances autophagy and is generally neuroprotective [105]. Lifestyle interventions that inhibit mTORC1 are also being investigated to arrest neurodegeneration [106]. As mentioned, amino acid deprivation inhibits mTORC1, reducing protein synthesis and stimulates autophagy. Nutrient rich conditions or basal neurotrophin availability reverse starvation-induced catabolic states, normalize homeostatic autophagy and promote neuronal survival [107]. Under neurodegenerative conditions however, inhibition of mTORC1 (e.g. by rapalogs or activation of AMPK), will stimulate autophagy and the removal of misfolded proteins [108, 109], including Aβ [110]. Mechanistically, these measures promote Atg transcription and recruitment to the phagophore by disinhibiting ULK-1/2 complex formation [111, 112].

## Alzheimer’s disease

AD is the most common form of neurodegeneration (60%), impacting 10% of the world's population over 65. The AD brain often features cerebrovascular pathology and is clinically overlapping or co-morbid with vascular cognitive impairment and dementia (VCID) in another 20% of cases [113]. AD is characterized by the accumulation of protein aggregates, namely amyloid β-peptide (Aβ) in the form of extracellular plaques and intracellular phospho-tau (p-tau) in the form of neurofibrillary tangles. The plaques become foci of an inflammatory reaction and neuritic dystrophy. These are associated with synaptic damage and neuronal loss, particularly in the hippocampal and the medial temporal/inferior parietal lobules regions of the brain, resulting in early memory dysfunction [114, 115]. Age is by far the greatest risk factor for sporadic Alzheimer's disease such that prevalence of AD per age group is 3% in 65-74; 17% in 75-84; and 32% in > 85 age range (www.Alz.org Facts and Figures 2020).

### Lifespan Extension

As AD and PD are age-related diseases, the relationship between mTOR inhibition and the extension of lifespan is relevant to discuss. Increased longevity, often associated with an increase in cognitive health span, has been achieved in several *in vitro* and *in vivo,* transgenic and wild type rodent, model systems via means that inhibit mTOR, including: 1) down regulation of insulin/IGF-1 signaling pathway [116, 117], 2) caloric restriction/SIRT1 stimulation [70, 118] and 3) mTOR inhibition with rapamycin treatment [119]. A particularly impressive study demonstrated that, in mice, carbohydrate restriction decreased mTORC1 activity, increased the resilience of memory function to ageing, and increased median lifespan by 13% [120].

The evidence for lifespan extension in mice fed rapamycin or primates placed on caloric restriction, even when implemented in adulthood was raised earlier. This sits favorably with the neuroprotective role of rapamycin in AD models, as will be discussed below. In contradistinction to the longevity research, there is a large body of evidence favoring the *upregulation,* or at least restoration, of insulin/IGF1 signaling for neuroprotection in symptomatic AD [121, 122]. Since insulin also activates mTOR, this may seem counterintuitive. However, as we consider AD and insulin resistance, as well as disease stage, inhibiting just mTORC1 may sufficiently reset insulin sensitivity.

### Insulin Resistance

After ageing, systemic insulin resistance and diabetes also present as risk factors for AD [123], resulting in an odds ratio (~2.0), almost on par with inheriting a single APOE4 gene allele [124–126]. In support of a causal relation, experimental diabetes is shown to drive AD pathology [127, 128]. It is also well accepted that the AD brain is itself characterized by a unique form of diabetes mellitus, so called ‘type lll DM’. The evidence points to a combination of insulin deficiency [129], attenuated insulin receptor expression [130] and insulin resistance [131, 132]. The mechanisms behind the insensitivity of the PI3K/Akt/mTOR pathway to the anabolic and neuroprotective action of insulin/IGF-1, are also multiple and include reduced ligand binding to cognate receptor (e.g. IR), IRS-1 deactivation or desensitization, as well as other more downstream signal transduction impairments [133–136]. Exposure to synthetic or expressed Aβ peptide is experimentally shown to result in each of these, for example demonstrating that Aβ can compete with insulin for insulin receptor binding [137, 138]. Disruption of insulin/Akt signaling may also be induced in the live brain by exposure to neurotoxic Aβ oligomers, as shown in monkeys [139]. This cerebral insulin resistance can result in decreased synaptic activity and density [1, 140]. Correspondingly, stimulation of the insulin/mTOR pathway downstream of insulin, using Akt or PI3K activators, has been shown to rescue synaptic density and plasticity in rodent models of AD [141, 142].

### mTORC1 in Alzheimer’s Disease and Down Syndrome: Hyperactivation

Numerous reports have linked alterations in mTOR signaling to age-dependent cognitive decline and to the pathogenesis of AD [143]. However, there are differing accounts of mTOR status in AD brain, transgenic mice and cell models. Several groups report dramatic up-regulation of basal (unstimulated) mTOR signaling markers in AD, mild cognitive impairment (MCI) and preclinical AD patients. These include increased p-Akt (Ser473), p-PI3K (Tyr508), p-mTOR (Ser2448), p-p70S6K (Thr389) and p-4E-BP1 (Thr37/46) over their respective total protein levels. One group took these as evidence for the general overactivation of the PI3K/Akt/mTOR signaling axis and made further correlations with decreased autophagy marker expression and increased inhibitory phosphorylation of IRS-1 [50, 51]. Similar abnormal activation markers have been found by several other groups [137, 144–151] (see Table 1). It is likely that these changes in mTOR activity are disease stage dependent. To illustrate, hyperactivation of mTOR is found in early to mid-stage AD brain by some [145, 178], but only in severely affected AD cases by others [152].

**Table 1:**
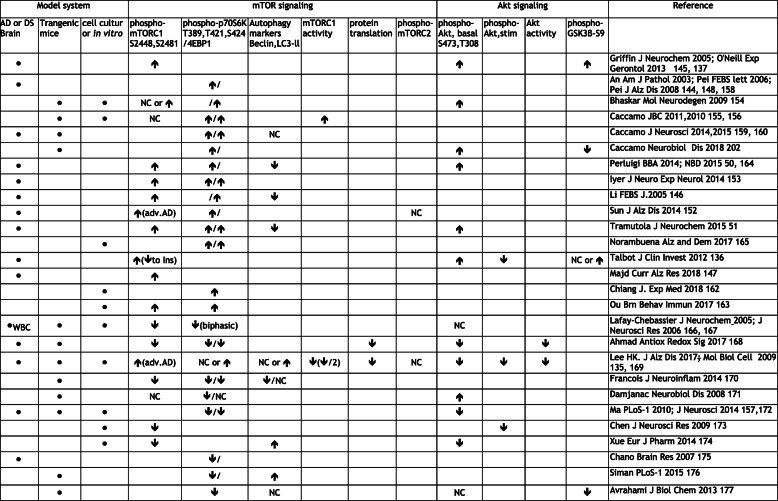
mTOR dysregulation in Alzheimer's Disease

Summary of the literature directly reporting on mTOR. The model systems employed by the various authors/laboratories referenced on the far right, are noted in the far left. 'Alzheimer or Down syndrome brain' may include other human cell types and either be post-mortem fixed, frozen or *ex vivo*. 'Transgenic mice' are rodent models that harbor human disease-causing mutations but include β-amyloid- or viral transgene-injections into wild type animals. 'Cell culture' refers to the use of primary neurons, immortalized lines, or mouse brain tissue slices that are transfected or exposed to β-amyloid, APP, tau or Presenilin as models of AD injury. Also included therein are other *in vitro* assays. mTOR signaling changes include phospho-mTOR, downstream targets phospho-p70S6K or p-4EBP-1, and enzymatic activity measurements as evidence for activation. The direction of change is noted, e.g. (↑) indicates hyperstimulation. Whether macroautophagy is initiated is also indicated. If data on Akt activation (p-Akt, insulin-stimulated p-Akt, downstream target phospho-GSK3β, or enzymatic activity is provided, a hyperactivation (↑) or inhibition (↓) is noted. A change qualified by context is noted by an 'or'. NC= no change. Most, but by no means all, studies favor hyperactivation of Akt and mTOR in various amyloid injury models, (top half of table) vs. inhibition (bottom half). Significant differences in model employed, stage of disease severity, time course, and other technical issues, account for the dichotomous findings (see text). Note that very few report on actual enzymatic activity assay. mTORC2 is relatively understudied in AD. See abbreviations. The compilation is illustrative only and not meant to be exhaustive. The authors apologize for any inaccuracies and unintended omissions

Table references are cited as : First Author, Journal, Year and reference number

The same hyperactivation of mTOR and Akt, as well as changes in autophagy markers and substrate p-p70S6K, are found in the Down Syndrome (DS) brain. These correlate with tau hyperphosphorylation [153]. The neuropathology of AD and DS similarly involves impaired mitochondrial function, increased oxidative stress, and proteostasis from derangements in the pathways that maintain the structural, quantitative, and functional stability of intracellular proteins [179]. The interconnected systems that govern protein homeostasis include the ubiquitin-proteasome system, autophagy network, endoplasmic reticulum, and mTOR pathway [180].

The molecular causes behind the autonomous basal activation of PI3K/Akt (and mTOR downstream) have not been completely worked out. PTEN is a protein tyrosine and lipid phosphatase that negatively regulates PI3K/Akt/mTOR by removing a phosphate from the essential lipid signaling molecule, PI3P (phosphoinositol 3,4,5 triphosphate) to generate PI2P. First, it is suggested that Aβ directly inactivates PTEN (phosphatase and tensin homolog) and thereby disinhibits PI3K [51, 154]. mTOR was found overactivated in PTEN knockout mice and levels of neurodegeneration protein markers accumulate in hippocampal synaptosomes [181]. Paradoxically, PTEN was also found to positively regulate neuronal insulin signaling in N2a cells. This was ascribed to its protein phosphatase action in preventing detrimental ERK activation. PTEN suppression increased hyperphosphorylation of Tau [182]. These reports contrast with data collected on both transgenic (PS1/APP 2X) and *in vitro* viral-mediated AD models, where PTEN inhibition actually rescued synaptic and cognitive (object location and fear conditioning) impairments [183]. The beneficial effect of the PTEN inhibitor was mediated by the stimulation of PI3K/Akt. Conversely, PTEN over-expression led to synaptic depression (decreased LTP, augmented LTD). Aβ peptides applied to primary hippocampal neurons induced the same synaptic defects and dephosphorylations of Akt and GSK3 by recruiting PTEN to dendritic spines where it becomes overactivated [183]. A similar contrast in PTEN involvement is reported in AD post mortem brain, finding decreased inactivated (phospho) PTEN in one report [184] and PTEN downregulation (and Akt hyperactivation) in another [145]. Interestingly, mutations in the PTEN-induced kinase-1 (PINK-1), a ubiquitin kinase participating in mitochondrial quality control, cause recessive early onset Parkinson's disease [185].

In addition to PTEN, the other major negative regulator of mTOR activation is AMPK (AMP-activated protein kinase), a master cell energy sensor that is stimulated (phospho-Thr 172) during low substrate stress. Activators of AMPK affect Aβ metabolism: resveratrol increases Aβ clearance by stimulating mTOR-sensitive autophagy [186] and quercetin reduces Aβ generation by AMPK-mediated downregulation of BACE-1 expression [187]. Aβ oligomers reciprocate by inhibiting AMPK activity and causing insulin resistance [188].

Next, the resistance to insulin/IGF action that characterizes AD brain has been mechanistically linked to the inhibitory feedback phosphorylations of IRS-1 (S616 and S636) by pS6K [136, 178]. Aβ has also been implicated in this phenomenon too by directly activating mTOR (and indirectly, mTOR target p70S6K) in studies using transgenic models [155, 156, 189]. Aβ enables the phosphorylation of PRAS40, an inhibitory subunit of the mTOR complex, thereby releasing mTOR activity [156]. The consequence is a decrease in IRS-1 levels [178, 190]. Here too there are conflicting reports, for instance a study in transgenic 2576 mice where Aβ is also co-localized to mTOR, but instead having an inhibitory role [157].

Actual insulin resistance was convincingly first demonstrated in post-mortem AD brain by Talbot et al [136]. Interestingly, it was the inhibited PI3K/Akt signaling response to insulin stimulation that was most impressively reduced (90%) in these viable human samples, even overshadowing the basal hyperactivated status of Akt and mTOR under unstimulated *ex vivo* conditions. As pointed out, the targeting of IRS-1 for phosphorylation may actually involve kinases other than mTOR/p70S6K [136]. Additional mechanisms of proximal insulin resistance include reduced numbers and activity of Insulin and IGF-1 receptors [178, 191].

### β-Amyloid

In cell models, using either transgenic primary cortical neurons (PCNs) or control PCNs exposed to Aβ oligomers, the abnormal hyperactivations of Akt (p-S473) and mTOR (p-S2448)/4E-BP1(p-S65) were associated with aberrant cell cycle reentry [154]. In addition to the cell culture studies, mTOR signaling was found to be abnormally upregulated after direct injection of Aβ oligomers into mouse hippocampus [156]. Basal (unstimulated) mTOR signaling increases are also described in triple (3x) AD and PDAPP transgenic mice. In these models, inhibition of mTOR with rapamycin rescued early learning and memory deficits and activated autophagy [156, 192]. In further experiments by the same group, intra hippocampal anti-Aβ antibody injections normalized the abnormal activation of mTOR. In their model, Akt hyperactivation was deduced to drive PRAS40 phosphorylation, thereby de-repressing mTORC1 [156]. Recently, either genetic suppression or anti-Aβ immunization corrected abnormally hyperactivated mTOR and Akt in transgenic APP mice [162]. Consistent with these findings, Metformin was reported to attenuate spatial memory deficits in double (2x)APP/PS1 mice by enhancing AMPK activation, leading to the reversal of abnormal hyperactivated mTOR [163].

Abnormal mTOR activation enhances both Aβ deposition (by inhibiting clearance) and possibly generation (indirectly, via insulin resistance, insulinemia and hyperglycemia) [137, 173, 192, 193] . Accordingly, either rapamycin treatment or AMPK-activation, by inhibiting mTORC1 and stimulating the autophagy machinery (Atg-1/Ulk), are shown to enhance Aβ clearance, reduce deposition, and abate pathology in transgenic AD mice [110, 192, 194–196]. Direct effects of mTOR pathway components on the α-secretase processing of APP (preventing Aβ generation) or β- and γ-secretase amyloidogenic activities has not been extensively investigated. The antidiabetic drug metformin activates AMPK, a negative mTOR regulator and stimulator of autophagy, promoted beta and gamma secretase cleavage activities and resulted in Aβ generation in SH-SY5Y cells and in an AD mouse model [197]. To the same end result, rapamycin treatment of APP-transfected N2a cells or transgenic AD mice resulted in enhanced Aβ production, but by inhibiting ADAM-10, an important α-secretase candidate [198]. Nevertheless, this area also requires more clarification as there is data pointing to an under-regulation of Rheb GTPase, a strong mTOR activator, that is correlated to elevated levels of BACE-1 in AD brain and where over-expression of Rheb reduced Aβ generation [199].

Finally, insulin impairments in transgenic AD mice were also found to be mTOR dependent. For instance, an improvement in central insulin dysregulation and reversal of impaired cognition was demonstrated when brain mTOR activity was genetically lowered by one copy in Tg2576 mice [161]. In another amyloid-based model with AD-like brain pathology, rats with T2DM and injected in the hippocampus with Aβ, revealed over-activation of the mTOR signaling pathway and suppression of activated AMPK. Rapamycin treatment produced a reduction of p-mTOR and partially restored p-AMPK levels, causing a reversal Aβ and tau deposition in the hippocampus and improvement in learning and memory [200].

### Tau

Regarding the tau pathology in AD, mTOR hyperactivation may also be responsible for hyperphosphorylation and cytoplasmic vacuolar collections of tau [201]. By acting on multiple Tau kinases (e.g. p70S6K) [158], as well as by inhibiting PP2A (the major Tau phosphatase), an overstimulated Akt/mTOR axis can drive tau hyperphosphorylation [144, 146, 158, 201–204]. The role of GSK3β (Tau kinase-1) in this context is however uncertain, since Akt, if stimulated by Aβ as hypothesized, would be expected to and does drive GSK inhibition (phospho-S9) [51]. In any case, activated mTOR marker levels positively correlate with neurofibrillary tangle (NFT) load and total- and paired helical filament (PHF)-Tau burden [144, 146, 205, 206]. Abnormal mTOR activity can also drive excessive Tau mRNA translation, via p70S6K [144]. Consistent with this, rapamycin (by inducing autophagy) retards cognitive decline and clears tau pathology [155, 207].

Notably, AMPK is also a tau kinase (Thr231 and Ser 396/404). Activated phospho-AMPK (p-Thr172) accumulates in tau tangle-bearing AD neurons and in other tauopathies [208]. Increased p-AMPK, as well as the indirect mTOR target, p-eEF2K (via p70S6K), were also demonstrated by Western technique in postmortem AD and 2x APP/PS1 transgenic mice brain extracts. This pathological hyperactivation of AMPK correlated with impaired LTP and was rescued by an AMPK inhibitor, but mTOR status itself was not tested [172]. The concurrent activations of mTOR and AMPK were both found in post mortem AD brain and co-localized with Tau pathology [147]. This is an interesting pairing given the likelihood of overactive AMPK to both inhibit mTOR and to directly phosphorylate eEF2K, a repressor of protein elongation by phospho-inactivating eEF2. These two actions would reduce *de novo* synaptic mRNA translation and inhibit LTP neural plasticity [209]. AMPK activation may also reflect a compensatory response. Recently it was discovered that expression of the AMPKα1 isoform is increased in post mortem AD hippocampus and in AD mice models. Brain specific repression of this isoform in model mice alleviated: cognitive deficits (novel object recognition and spatial learning and memory in the MWM), restored hippocampal LTP, improved spine morphology and blunted the abnormal inhibitory hyperphosphorylation of eEF2 due to overactive AMPK, thereby increasing de novo synaptic protein synthesis [210]. These studies may paint a consistent picture of abnormal AMPK activation in AD, but it remains unproven if either AMPK or PTEN activation attenuate or aggravate AD pathology [211].

In a Drosophila Tauopathy model, mTOR activation was found to mediate cell cycle reentry and neurodegeneration [212] and blocking mTOR signaling rescued Tau-mediated toxicity in such flies [213]. The same neuroprotection was afforded by rapamycin in tau transgenic mice [202, 214] and in mice stereotactically injected with AAV-hTauP301L into the hippocampus [176]. Suppression of mTOR with rapamycin thus mitigates both Aβ and tau pathologies.

### Autophagy

In addition to the insulin signaling derangement, the autophagy system of protein disposal and recycling is altered in AD [51, 215]. Autophagy is a major clearance mechanism for Aβ in neurons, working alongside microglia, the Ubiquitin Proteasome System (UPS) and amyloid degrading enzymes [216–218]. In the early stages of AD or in animal model brains, autophagic vacuoles accumulate in dystrophic neurites. However, rather than primarily caused by changes in mTOR, it appears to come about primarily because lysosomal acidification, autophagosome fusion and/or clearance are reduced, resulting in net impairment of autophagic flux [194, 215, 219]. Nonetheless, inductions of autophagy-related protein Atg5, Beclin-1 and ULK-1 probably play a role in Aβ degradation, as demonstrated using a small molecule rapamycin enhancer or starvation in APP expressing N2a cells [108]. Autophagy marker levels (Beclin-1, LC3) were found decreased in mild cognitive impairment (MCI) and AD brain. This loss of autophagy correlated negatively with amyloid load and was associated with a hyperactivated PI3K/Akt/mTOR axis [51]. Consistent with these observations, the suppression of mTORC1 by rapamycin, induces autophagy flux and ameliorates cognitive deficits in transgenic mice [220]. Genetic reduction of mTORC1 in Tg2576 AD mice also reduced Aβ pathology, stimulated autophagy and rescued memory deficits [160]. Abnormal p-tau levels and pathology can also be cleared by mTOR-dependent autophagy [112, 221], an outcome similarly observed in 3x transgenic AD mice treated with rapamycin [155, 192]. Not surprising, p-Tau in turn may also impair autophagy [222].

### Synaptic Protein Synthesis

Complementing mTOR's role in suppressing protein removal, is its positive regulation of mRNA translation and protein synthesis at synapses. Ribosomes and mRNA are transported from soma to dendritic spines where mTORC1/2 are active [7, 10] and have an important role in plasticity and learning [9, 223]. One example of a TOP (5' terminal oligopyrimidine)-mRNA that is translated in dendrites following LTP induction and mTOR activation is the elongation factor protein eEF1A [224]. Other mTOR-dependent, rapamycin- sensitive, specific synaptic target proteins driven by activity or BDNF/Insulin stimulation include NMDA-R, CamKllα, PSD-95 and Arc [96, 225, 226]. How then is protein synthesis affected in the various neurodegeneration syndromes? The purported hyperactivity of mTOR in AD would be expected to result in excessive and detrimental synaptic protein synthesis. Genetic examples of this phenomenon include tumor growth in Neurofibromatosis 1 and Tuberous Sclerosis, wherein PI3K/Akt and mTOR are upregulated, respectively. Moreover, cognitive deficits and autism define both illnesses [227, 228]. The identity of the excessively translated mRNAs is however not yet clarified. Another example of mTOR-dependent synaptic protein synthesis and phosphorylation is the loss of function of the translational repressor, FMR protein, in the Fragile X syndrome - also defined by mental retardation and autism [229, 230]. The derepressed translation of mRNA results in excessive proteins synthesis [231]. Returning to AD, despite reports citing overstimulation of mTOR, activity-dependent synaptic translation was impaired, not increased, in both an AD mouse model and in post-mortem AD brain. ROS-mediated damage to Akt and mTOR in the signaling pathway was the cited reason [168]. It remains for future research to determine if the mTOR-dependent production of other plasticity relevant proteins in dendritic spines such as NMDA-R1, αCaMKll, CPEB, and Arc [232] are affected in any of the neurodegenerative conditions.

### Vasculopathy

Briefly, vascular dysfunction in AD is characterized by chronic hypoperfusion, blood brain barrier disruption, reduced vascular density and reactivity and impaired neurovascular coupling. Here too, unchecked mTOR activity has a deleterious role, in part by inhibition of NOS and decrease in NO (nitric oxide) bioavailability [233]. Transvascular Aβ clearance is also reduced. mTOR attenuation by rapamycin in hAPPJ20 AD mice can accordingly lessen these changes [234].

### mTORC1 in Alzheimer’s Disease and Down Syndrome: Hypoactivation

In contrast to the aforementioned reports concluding hyperactivation of mTOR in various AD models, a substantial number of groups point to no change or even down-regulation of mTOR signaling, as well as neuroprotection from actually stimulating mTOR. These also deserve mention for balance (Table 1). For instance, in a recent study of autopsy brain, levels of p-mTORC1 (S2448 or S2481) and of total mTOR, revealed no statistical differences across the clinical groups (AD vs. control) [235]. In PDAPP mice, there is no reported difference in mTOR target p-p70S6K levels between untreated transgenic and wildtype mice [192]. On the other hand, p-mTOR (pS2448) and p-p70S6K were reduced in N2A cells affected by aggregated Aβ42 treatment, in 2x transgenic APP (sl)/PS1(M146L) mouse cortex and in AD lymphocytes, compared to controls [166, 236] . Moreover, APP (swe)/PS1(deltaE9) 2x transgenic mice display increased autophagic activity accompanied by decreased mTOR activity [237]. In yet another 2x model, APP (sl)/PS1(KI), mTOR itself was unchanged but downstream activation of p70S6K (pT389) was reduced rather than stimulated [171]. Consistent with these studies, but using a growth factor stimulation paradigm in rat PCNs, Aβ treatment inhibited BDNF-induced Akt/mTOR signal activation [173]. Similar inhibition of neurotrophin-stimulated Akt/GSK3β-S9 phosphorylations were found in N2a cells exposed to oligomeric Aβ-containing fractions obtained from 2x AD transgenic mouse brain [238]. In the presymptomatic Tg2576 model, an early impairment of long-term potentiation (LTP) was correlated with inhibited mTOR signaling (lowered p-p70S6K and p-4E-BP1), similar to results in wild type brain slices exposed to either Aβ peptide or rapamycin [157, 239]. In the same model, up-regulation of mTOR rescued LTP [170]. The role of systemic insulin resistance in modifying mTOR signaling in AD was recently probed using two rat models; ('T2DM': intraperitoneal streptozotocin (STZ) on high fat diet and 'AD': hippocampal Aβ injection). In comparing the Control, T2DM, AD and T2DM+AD animal groups, total mTOR protein and mRNA levels in the hippocampus as well as the phosphorylation of tau protein were significantly increased only in the combined T2DM+AD group, not in the AD alone group compared to control [240]. How the sustained mTOR hyperactivation phenotype required concurrent Aβ toxicity and systemic insulin resistance is not clear. An intracerebral STZ-induced AD rat model evidenced reductions in all of Akt, IRS, p70S6K and mTOR, but p-mTOR was not tested [241]. Inhibited mTOR activity (p-p70S6K1), reduced fear conditioning memory and plaque pathology each characterize the 5X-transgenic AD mouse model. These defects were all rescued with an inhibitor of GSK3β, providing a novel mechanism to restore mTOR activity, reduce autophagy and improve lysosomal acidification. Tau pathology was not reported [177]. Finally, the reduction in mTOR signaling and basal phospho-Akt marker levels, as well as enzymatic activities, in synaptosomes from 2xAPP/PS1 mice and post-mortem AD brain, was correlated with inhibited BDNF-stimulated protein translation. Oxidatively damaged synaptic Akt was held responsible and Akt enhancement rescued protein translation [168]. The role of oxidative stress in AD-associated insulin resistance is elsewhere reviewed [242]. The observation that Aβ may stimulate AMPK, perhaps a compensatory effect, may partially explain the reduction in mTOR activity observed in some of these studies [135, 195, 243].

### mTORC2 in Alzheimer's Disease

Some studies have started to look separately at mTORC1 (Raptor) and mTORC2 (Rictor). One group found neither total- nor phospho-mTOR levels (nor specific total and p-Raptor of mTORC1) were significantly changed in early to moderate AD hippocampus compared to control. p-mTORC1 and p-Raptor was however significantly increased in severe AD. The same work reported that Rictor (of mTORC2) levels were unaltered in AD [152]. In our work, both total mTORC1 and 2 (rictor) levels and respective enzymatic activities were reduced in advanced AD brain and transgenic models. Autophagy markers were increased and protein synthesis was inhibited [135]. Nevertheless, phospho-mTOR / total-mTOR was increased and we also found that application of rapamycin, by further reducing mTORC1, was cytoprotective. Interestingly, overexpression of Rictor was similarly beneficial. A proteomics study of neural cells expressing wild type mTOR also concluded upregulation of C2, but not C1, increased cell viability by facilitating pro-survival and suppressing caspase-mediated apoptotic genes and by stimulating p-Akt (Ser473/Thr308) [244]. These results in AD models are consistent with mTORC2 survival promoting functions [245, 246].

In conclusion, we noted AD models in which baseline mTORC1 is abnormally over-activated and other models in which markers of activation (e.g. p-p70S6K) are either unchanged or reduced. One obvious reason for contradictory findings reported by various laboratories in the activation state of mTOR and signal kinases in general is that the various disease models and/or assays may not comparable. The upstream factors that negatively control mTORC1 activation such as PTEN, AMPK and TSC1/2 (also a positive regulator of mTORC2 [71] are not always assessed but may themselves be the proximate cause of variation between cell lines and models [87]. Another is that attention to both basal conditions and activation testing under neurotrophin stimulation is not always undertaken. A third is that phosphoprotein levels alone may not always be a proxy for actual enzyme activity in certain situations. An example of dissociation is that mTORC2 and PI3K can maintain Akt phosphorylation (perhaps compensatory) in the presence of a pharmacologic inhibitor of Akt activity (resulting in disruption of downstream GSK3β phosphorylation) [247]. There is also the disease stage and/or time course of experimental perturbation that needs to be controlled. For instance, the regulator of mTORC1 and target of C2, Akt (as well as its substrate GSK3β) undergo a biphasic, age dependent change in phosphorylation in PS1xAPP transgenic mice hippocampus (6 mo.-Akt activation vs. 18 mo.- Akt inhibition). This depends on the ratio of soluble APPα and oligomeric Aβ along the disease time line [238]. Duration of Aβ exposure showed similar biphasic results in primary neurons. With aging, changes in NMDA- and α-nicotinic ACh-receptors were implicated in biphasic opposing directions of Akt status [248]. Paradoxes such as rapamycin inhibiting mTOR-dependent synaptic plasticity- yet is neuroprotective in the various AD models (see below)- may find explanation in signal feedback and crosstalk complexity. For instance, rapamycin, while inhibiting mTORC1, can also induce Akt phosphorylation with overriding beneficial actions (in addition to the stimulation of autophagy). It does so by inhibiting p70S6K-T389 phosphorylation, thereby stabilizing IRS-1 [249, 250].

Regardless of the contradictory reports, most agree that the response of the Akt/mTOR axis to neurotrophin/insulin stimulation is suppressed in AD, consistent with a state of insulin resistance, and that mTORC1 inhibition with rapamycin is neuroprotective, reduces proteinopathy and actually restores memory formation and maintenance [155, 192]. Conversely, stimulation of mTORC2 might be beneficial. This underscores the duality of mTOR roles in health and disease with respect to synaptic plasticity [251].

## Parkinson’s Disease

Sporadic PD is the second commonest neurodegeneration after AD. Aging is again the primary risk factor, as it affects 1-2% over age 65. Here mTOR also emerges as a novel therapeutic target [81, 252]. There is a large body of evidence that mTOR is perturbed in PD models [253, 254]. Perhaps more so than AD, oxidative stress (ROS) is a major contributor to the selective degeneration of dopaminergic neurons in PD [255]. A major source of ROS are the mitochondria in PD that are deficient in electron transport Complex 1 activity. In the MPTP-treated mouse model of PD for instance, mitochondrial ROS are shown to stimulate apoptosis [256]. Various other neurotoxins (e.g. ceramide, rotenone, H2O2, 6OH-DA, paraquat) are also used to model PD pathophysiology. In general, these manipulations suppress mTOR/Akt activity and restoring mTOR functions by overexpressions of either the wild type form or p70S6K rescues neuronal death in these models [254, 257]. In alignment with this view, rapamycin predictably potentiates the oxidative stress [258, 259]. One mechanism of ROS-mediated inhibition, at least as uncovered from using AD-affected synaptosomes, is oxidative damage to Akt/mTOR signaling enzymes, resulting in the functional loss of activity-dependent protein translation [168].

A significant body of data implicates pathologic induction of the gene for REDD1/RTP801 protein in Parkinson's disease, the only function of which as a regulator is to suppress mTOR [253]. Up-regulation of RTP801 is shown in various PD neurotoxin cell models (6OHDA, rotenone and MPP+) as well as in post mortem PD substantia nigra neurons. RTP801 experimentally promotes cell death (protected by knock-out) via an interaction with TSC (relieved by TSC shRNA) to inhibit mTOR function (reducing p-mTOR and p-p70S6K levels). The TSC-2 requirement was confirmed [260]. Although mTOR activation status in PD brain was not reported on, it is clear that activation of the cell survival kinase Akt (phospho T308 and S473) was inhibited in PD brain and experimentally reproduced in cells. Constitutive Akt expression also protected PC12 cells from either RTP801 or 6OHDA) [261]. The additional mechanism advanced to explain this phenomenon is that REDD1 also blocks mTOR-dependent phosphorylation of Akt. Accordingly, mTOR overexpression protected cells from 6OHDA toxicity. Other examples of mTOR over-expression as beneficial to correct the deficiency were in an AD model cited earlier [157] and in a HD model discussed below.

On the flip side, there is a PD mouse model, where mTOR/Akt is upregulated 1 week out from a single injection of MPTP and autophagy markers proportionately reduced [262]. The nuanced role of mTOR was further elaborated using this model and confirmed *in vitro* by showing that the mTOR pathologically upregulated protein translation to toxic levels. Correspondingly, rapamycin proved neuroprotective by correcting this and restoration of Akt signaling [263]. Apparently, rapamycin also specifically blocks REDD1 protein synthesis, and so maintains Akt phosphorylation [263]. Interestingly, REDD1 is reported to be the target of metformin to inhibit mTOR, rather than AMPK [264]. Treatment with temsirolimus *in vivo* induced autophagy and maintained high Beclin-1, p62, and MAP (microtubule-associated protein) 1A/1B-light chain 3 (LC3) expressions, while inhibiting p70S6K expression in another MPTP model of PD [265]. Still others have found that inhibition of mTORC1 signaling also revert cognitive and affective deficits in a 6OHDA mouse model of PD [266].

Recently, the complex regulation of mTORC1/raptor and C2/rictor activations were examined in neuroblastoma cells and wild type mice either exposed to or injected with the mitochondrial toxin rotenone, respectively [267]. Under full serum and dietary nutrient conditions, rotenone activated mTORC1 and inhibited mTORC2/rictor, compared to baseline. These results indicate these two complexes, preferentially controlling cell growth and survival, respectively, are reciprocally regulated, both in the neurodegenerative context and depending on nutrient levels.

It is important to note that accumulation of α-Synuclein and Lewy body-like formations, the proteinopathy hallmark of PD, is generally lacking in 6OHDA- and MPTP-, but present in rotenone -ROS-generating models of PD such as the one just cited above. Genetic models of PD also generally do not form Lewy bodies, but the clear exception is transgene wild type or mutant α-Synuclein. α-Synuclein modulates synaptic activity and point mutations in the SNCA gene cause rare, early onset autosomal dominant PD [268]. Deficient autophagy may contribute to α-Synuclein accumulation in PD or Lewy Body Dementia and stimulation of autophagy by the mTOR inhibitors rapamycin or everolimus may promote its clearance [269–271].

**Fig. 3:**
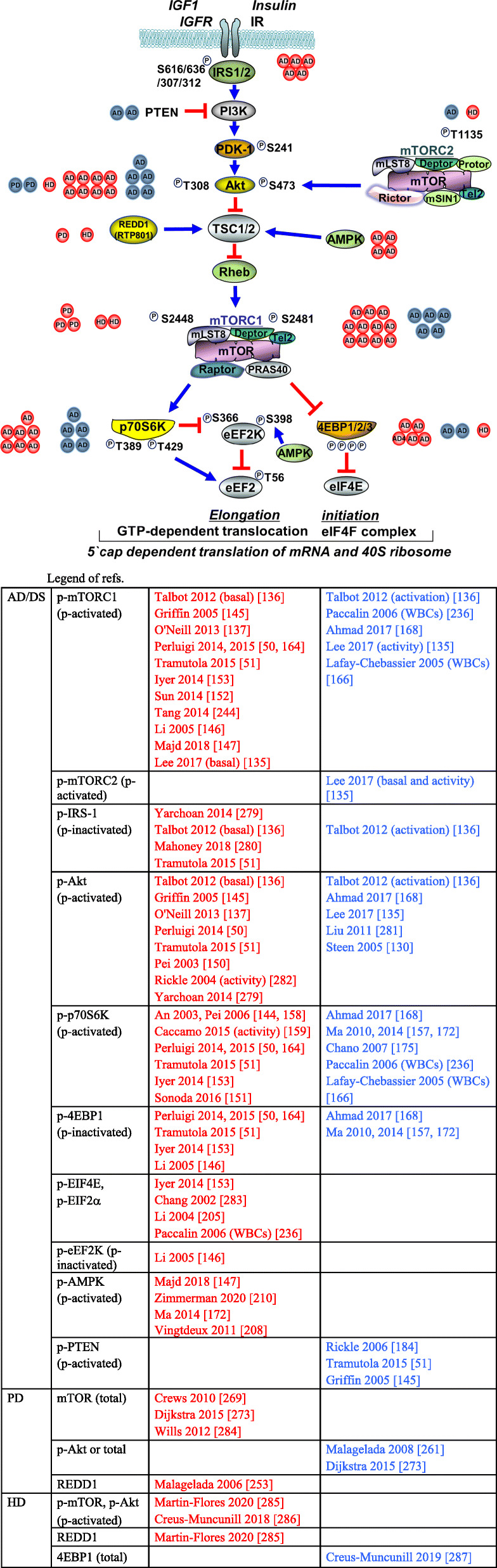
mTORC1 pathway and regulatory protein changes in human brain. Relative levels of phosphorylated forms and activity status of several major mTOR pathway components and regulators (PTEN, mTORC2, REDD1, AMPK) in neurodegenerative disorders. Only those studies that examined human AD/DS (Alzheimer's, Down's Syndrome), PD (Parkinson's), HD (Huntington's) or ALS (Amyotrophic Lateral Sclerosis) brain or peripheral cells (WBCs) are listed. In addition to those studies cited in Table 1, that examined mTOR pathway proper, we include those here that did not but still focus on one or more of the other components. In so far as the number of studies supporting a given direction of change (references reporting decreased levels in blue, increased in red, in affected vs. control brain) can be taken as some measure of consensus, each publication listed is represented visually by a single red (increased) or blue (decreased) dot next to the respective pathway protein. The dot also indicates the corresponding disease. Most studies only examined basal phospho-levels, whereas a few specified insulin-induced activations or assayed the enzymatic activity. For example, the majority of studies in AD brain favor *basal* mTORC1 over-activation (vs. inhibition; n=11 vs. 5); increased IRS-1 phospho-inhibition (5); AMPK over-activation (4) and increased 4EBP1 phospho-inhibition (5 vs. 2), whereas a more modest majority, favors Akt over-activation (8 vs. 5) and p-p70S6K hyperactivation (7 vs. 5). Nevertheless, the Akt and mTOR activation responses to insulin are depressed in cell model [135] and AD brain [136]

In PD brain, as in AD, mTOR also appears upregulated and autophagosomes accumulate [272, 273] (Fig. 3). In neuronal cultures and mice expressing mutant A53T α-Synuclein, mTORC1 signaling is overactivated, also resulting in insulin resistance (via IRS-1, S636 phosphorylation). The changes were reversed by rapamycin [274, 275]. Metformin was also found to clear cytoplasmic α-Synuclein in hippocampal neurons, as did rapamycin, by inhibiting mTOR, but interestingly not via AMPK or autophagy induction [276]. The ubiquitin hydrolase UCHL1 and PINK-1 proteins both activate mTORC2, predicting improved cell survival [277, 278]. The role of mTORC2 in PD clearly bears further investigation.

Figure 3 Legend of refs. [50, 51, 130, 135-137, 144-147, 150–153, 157–159, 164, 166, 168, 172, 175, 184, 205, 208, 210, 236, 244, 253, 261, 269, 273, 279-287].

Interestingly, several familial PD-linked proteins, affected by disease-causing recessive mutations in PINK-1/ PRKN (Parkin) and DJ-1 (PARK7) genes and in the dominantly inherited LRRK2 gene, influence the autophagy-lysosomal pathway in response to mitochondrial damage in an mTOR-independent manner [288, 289]. Adding to the complicated story of mTOR in PD noted above, these studies have generated interest in stimulating autophagy by means other than rapamycin and analogs in order to improve α-synuclein removal, i.e. targeting mTOR-independent autophagy. These strategies employ agents such as curcumin and Trehalose [290–292]. Whereas mTOR negatively regulates TFEB (transcription factor EB) to suppress autophagy, Trehalose acts on the Foxo-1 transcription factor to enhance autophagy protein expressions [293]. In light of this, the *in vivo* activation of autophagy through a combination of rapamycin and Trehalose treatment was shown to reverse both neuronal dopaminergic damage and behavioral deficits. Therefore, a dual therapy approach aimed at autophagy seems to hold promise for PD-like pathology [294].

## Huntington’s Disease

Huntington's disease is another dominantly inherited proteinopathy, resulting in a degenerative movement, psychiatric and cognitive disorder. Several reports clearly implicate abnormal synaptic plasticity, spatial memory cognition and dendritic spine loss early on in experimental mutant Huntington (mHtt) bearing mice [295–297]. In transfected cells bearing aggregating proteins with polyglutamine (polyQ) expansions, such as caused by the Huntington's disease mutation in which the CAG tract in the Htt gene is expanded or by mutant Ataxin 1 in the case of spinocerebellar ataxia 1, blockade of mTORC1 with rapamycin or pan-mTOR catalytic inhibitors results in stimulated autophagy followed by removal of mutant protein aggregates and cytoprotection [298, 299]. Drosophila and mouse HD models also benefited from mTOR inhibition [300]. In a polyQ htt mouse model, deletion of TSC1 led to activation of mTORC1, accelerated motor incoordination and premature death. In striatal cells overexpressing the same mutation, mTORC1 activation was induced which then could be abrogated by knocking down Rheb [301]. The authors conclude that enhanced mTOR is pathogenic in HD. In neuroblastoma cells induced to express mutant Htt polyQ72 fragments, catalytic inhibitors of total mTOR or mTOR specific siRNA, induced autophagy and reduced protein aggregates [298, 299]. Each of p70S6K, p4E-BP and p-Akt, downstream substrates of mTORC1 and C2, respectively, showed appropriate inhibitions. Still puzzling, everolimus, an allosteric mTOR inhibitor, had no effect [298, 299].

In an interesting application using a Drosophila model HD model, Lithium was used to activate mTOR-independent autophagy. This along with co-treatment with rapamycin to limit the undesirable side effect of GSK3β-mediated mTOR activation, resulted in enhanced mutant Htt clearance [302, 303]. But here too, there is evidence to the contrary in another rodent model of mutant-Htt, wherein mTOR activity was impaired. Reconstituted mTOR activation by constitutive Rheb proved cytoprotective [304]. In post mortem HD putamen, mHttQ111 transgenic mice, and in mHtt-bearing rat primary neurons, REDD1 (RTP801) is also upregulated and mediates cell death, as the case in PD [285]. Accordingly, downregulation of RTP801 prevented motor learning deficits in the mice. However, contrary to PD, Akt was hyperactivated, from increased Rictor action, invoking a compensatory effect. The activated Akt pattern was also confirmed by others in genetic mouse models of HD [305, 306]. One study points to a scenario where further increasing Rictor in striatal cells actually prevented neurodegeneration from mHtt expression [286]. In this mouse model and in HD putamen, rictor, Akt and mTOR activations were already increased, again pointing to a partially effective compensatory reaction. The same authors found evidence for excessive *de novo* protein translation in genetic HD mice, attributed to an increase in phosho-inactivation of 4EBP1. This hypofunction of 4EBP1 was confirmed in human HD putamen specimens, however it was left unclear if excessive mTOR was culprit [287]. In a forementioned study, RTP801 silencing proved to normalize the Akt hyperphosphorylation by reducing Rictor and enhancing synaptic protein synthesis [285]. Although rapamycin was not tested in these last 3 citations, the HD-PD movement disorders axis demonstrates the duality of PI3K/Akt/mTOR pathway involvement in various neurodegenerations and the need to tailor treatment if this is to be targeted.

## ALS and FTD

In another expansion mutation, a hexanucleotide repeat in the C9ORF72 gene causes the most common form of inherited ALS and FTD. The loss of protein function encoded by this gene promotes TDP43 accumulation in ubiquitin-containing inclusions. In a C9ORF72 knockout model, autophagic flux is increased and correlated with reduced mTOR activity (less p-p70S6K1). Hence, C9ORF72 protein is postulated to act as a negative autophagy regulator, perhaps in synergy with the binding of mTOR at the lysosome membrane [307, 308].

In a model incorporating another ALS-causing gene mutation (G93A in SOD-1), autophagy markers were also increased in spinal motor neurons [309]. Using this model, a small molecule that enhanced mTOR and suppressed autophagy suppression was found to be neuroprotective [310]. Moreover, Rapamycin actually accelerated disease progression [311, 312]. Interestingly, when mTOR-independent autophagy was activated with Trehalose, motor neuron lifespan was prolonged and protein aggregations were reduced. Therefore, in the context of these models, as contrasted with AD, motor neuron viability appears dependent on mTOR activity and autophagy needs boosting by other mechanisms [311, 312]. Progranulin (GRN) mutations resulting in haploinsufficiency also cause familial FTD, and in GRN genetic models Trehalose, is also found to be neuroprotective [312].

Another FTD transgenic mouse in which TARDP43 overexpression yields TDP43/ubiquitin containing inclusions, produced a different result. In this case, rapamycin treatment and mTOR inhibition-autophagy activation (LC3-1/LC3-II) proved neuroprotective against memory loss and inclusion formation [314]. Finally, in a mouse FTD model bearing a tau mutation in which the observed mTOR overactivation is associated with Tau accumulation and hyperphosphorylation, rapamycin also corrected behavioral deficits and afforded neuroprotection [202]. Based on these latter reports, a phase 2 clinical trial of rapamycin in ALS is ongoing [315].

## mTOR-based treatment

**Fig. 4:**
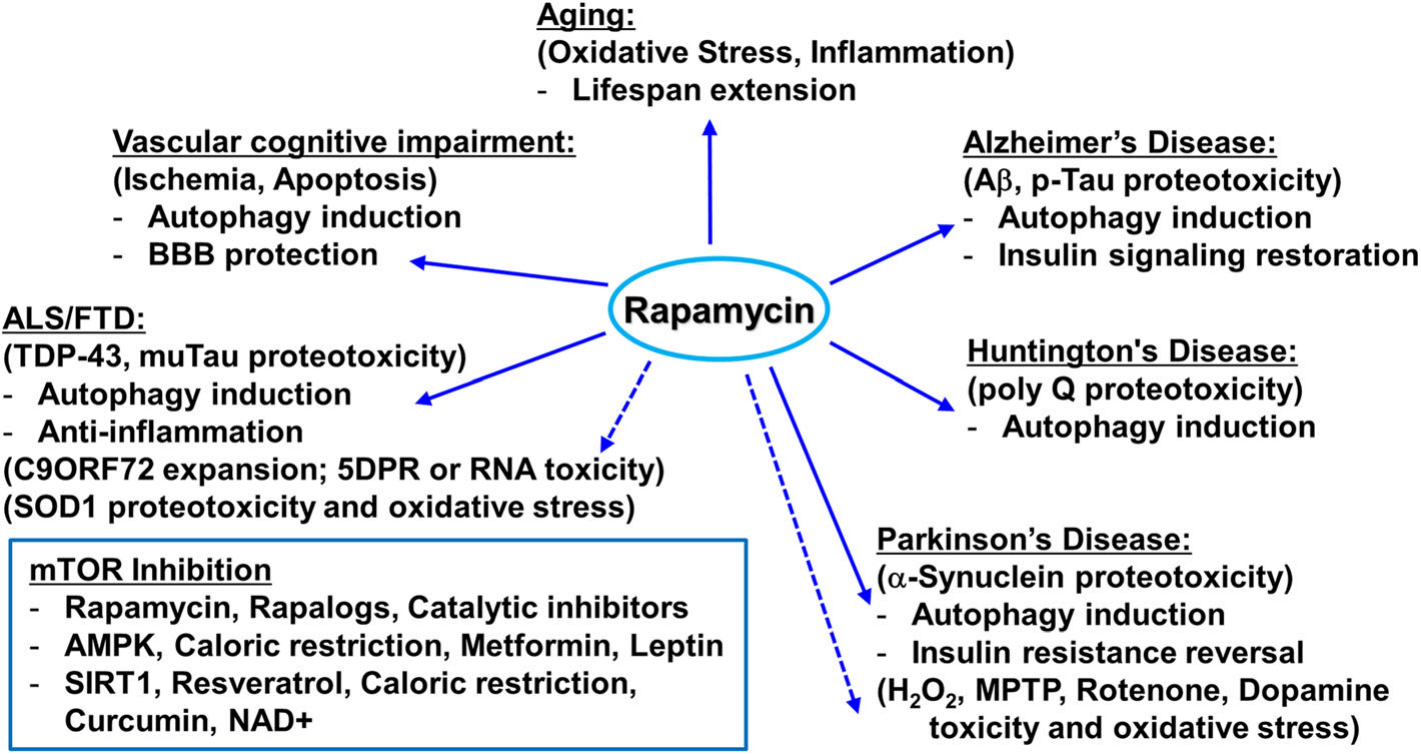
mTOR inhibition in neurodegenerative disorders. As a nutrient sensor, mTOR has important homeostatic functions to regulate energy metabolism and support neuronal growth and plasticity. However, in Alzheimer’s disease (AD), mTOR alternately plays important pathogenic roles by inhibiting both insulin signaling and autophagic removal of beta amyloid and Tau aggregates. Overactive mTOR also abets the cerebrovascular dysfunction of AD. Some of the other neurodegeneration conditions, discussed herein, have similar proteotoxic mechanisms (indicated in parentheses). The beneficial actions of mTOR inhibition with rapamycin are shown as arrows to the corresponding bulleted effects. Dashed arrows indicate unproven actions on those proteotoxic processes

Rapamycin (sirolimus), the prototypical mTORC1 inhibitor, is an immunosuppressant and anti-proliferative FDA-approved agent for kidney transplantation, coronary stents, cardiac hypertrophy and renal cell carcinoma [316]. It binds FK506 and allosterically stabilizes raptor-mTOR in a kinase-inactive complex. The discovery of the apparently paradoxical protective action of rapamycin in many models of neurodegeneration (see below and Fig. 4) arose from the early recognition that mTOR transduces the action of insulin and IGF-1 via Akt in both the periphery and brain. Other trophic factors (EGF, BDNF) also require some mTOR activity to promote enable their neuroprotective and cell survival functions [20, 317]. Moreover, the AD brain is intrinsically insulin resistant and does not metabolize glucose properly where needed. There is also a complex relationship to insulin resistance in the periphery since systemic T2DM doubles the risk for AD [126]. Therefore, much effort is devoted toward the development of anti-diabetic drugs to treat AD that include metformin, glimepiride (a sulfonylurea), GLP-1 and Liraglutide (a glucagon-like peptide analog) and intranasal insulin [318, 319]. These strategies appear to enhance the Akt/mTOR signaling axis. The resistance to insulin signaling that characterizes AD brain would further predict that restorative mTOR activation would be neuroprotective. Therefore, a balanced treatment of the matter relating to the pros of rapamycin therapy should include the other instances where mTOR *activation* is favored.

Indeed, there are disease states, AD aside, where direct mTOR activation is neuroprotective. These involve CNS models of ischemia, trauma and oxidative stress and is attributed to the inhibition of apoptosis or repression of autophagy [259, 320, 321]. In one example, the cytokine and hormone erythropoietin that signals through mTOR activation, is regarded to prevent neuronal apoptosis during oxidative stress or hypoxia [322] and Aβ exposure [323]. Relating to Parkinson’s disease, an mTOR activating protein protected dopaminergic cells from H2O2 mediated oxidative stress [259]. REDD1 (RTP8011) is an endogenous mTOR inhibitor that is increased in dopaminergic neurons and contributes to neuronal death, mTOR activation is protective in this model too [253]. A number of physiological studies also support the notion that mTOR activation may counter neurodegeneration. mTORC1 control over activity-related 5' TOP mRNA translation initiation (via 4EBP1 and p70S6K phosphorylations) and dendritic protein synthesis is critical to synaptic plasticity (LTP and LTD) and memory formation [324, 325]. Rapamycin is accordingly found detrimental to normal synaptic plasticity by many laboratories [6, 7, 10, 95, 96, 326]. In agreement with this, is the loss of mTORC1 signaling found in two AD animal models (transgenic and wild type exposed to exogenous Aβ42) or in wild-type mice treated with rapamycin, causing impairment of late phase hippocampal LTP (and LTD) [157]. In behavioral correlates of activity-dependent synaptic strengthening, early studies show activations of mTORC1 and p70S6K during the consolidation phases of both spatial (Morris water maze) and fear conditioning paradigms. These long-term memory processes were understandably inhibited by either rapamycin or AMPK stimulation [8, 20, 95]. mTORC2 is also necessary for synaptic plasticity, perhaps via association with polysomes or cytoskeletal protein polymerization [223, 327]. These considerations auger for mTOR stimulation and by the same logic, against rapamycin treatment for AD.

However, it is the inhibition of mTORC1, downstream in the insulin pathway, with Rapamycin that consistently increases lifespan in mammals [13], rescues several forms of neurodegeneration [328], mitigates synaptic/neuronal losses and restores synaptic plasticity and/or cognition in several animal and cell disease models (summarized in Fig. 4). As we have seen for instance, rapamycin prevents loss of learning and memory in the Morris Water Maze in several AD mice models when given at young (2 mos.) or mid ages (4-7 mos.) [155, 192, 234, 329]. Perhaps surprising given its aforementioned negative effects on long term synaptic plasticity and memory, lifelong treatment with rapamycin even improved spatial memory in 2-4 mo. control and wild type mice [5, 189, 192]. However, neither LTP or spine morphological changes were assessed. A clear example of this principle is the TSC2 haplodeficient mouse, in which cognitive deficits are directly linked to hyperactivation of mTOR; rapamycin restores synaptic plasticity and cognitive function [330]. The excessive activation of mTOR associated with AD progression, as found in many studies and human brain samples (but by no means all, see Table 1, Fig. 3), also favors rapamycin-based therapy for the disease context [331]. In this regard, a major effect of rapamycin is to decrease proteotoxic aggregates, such as Aβ42 [332] via autophagy/lysosome induction [119]. Rapamycin/Temsirolimus appear beneficial in alleviating proteotoxicity in several transgenic AD [155, 192, 234, 329] and tauopathy mice strains [176, 202]. Cellular models under toxic stress from β-amyloid [333] and other aggregate-prone proteins are also alleviated by rapamycin [298]. In Parkinson disease cell-based and transgenic models where α-synuclein accumulation is proteotoxic, mTOR inhibition with rapamycin and/or autophagy induction with Beclin or Atg7 were cytoprotective [269, 334]. In the latter reference, total and phopsho-mTOR levels were increased in DLB and α-synuclein transgenic brains. However, the concept of rapamycin rescue may hold regardless of the basal activation status of mTORC1. In certain HD drosophila and mice models for instance, although basal mTOR activity is already downregulated, further inhibition with rapamycin stimulated autophagy, cleared polyQ Htt protein fragments and was cytoprotective [300]. This scenario applied to some AD models as well [135]. Mixed pathologies are also amenable to therapy. Examples include rats with experimental hippocampal AD pathology on a T2DM background [200] and vasculopathy with blood-brain barrier breakdown in transgenic hAPPJ20 mice [335]. In one instance, rapamycin was even found helpful in protecting hypoxic primary cortical neurons from apoptosis by stimulating autophagy [336]. Another caveat is that once the neurodegeneration is too advanced, rapamycin may become ineffective [220].

One conclusion is that where toxic proteins accumulate in neurodegenerative disorders, the advantage of autophagy induction by inhibiting mTOR may outweigh the antiapoptotic and pro-synaptic effects of its activation. As well, the effects of mTOR inhibition in normal tissue studies doesn't have to coincide with its effects in the various disease states. This situation could possibly arise from crosstalk between C1 and C2 circuitry [87]. It seems likely therefore that these two mTOR activities are differentially altered in neurodegenerative conditions, arising from changes to the gain of their positive regulators and negative feedback loops (e.g TSC1/2, PI3K, p70S6K). Underscoring the fine balance in their circuitry, both chronic activation of mTORC1/p70S6K [71, 337] and conversely, prolonged rapamycin treatment [90], can each result in mTORC2 inhibition and lead to insulin resistance.

## Other mTOR strategies

Unfortunately, rapamycin has a systemic toxic profile that includes pneumonitis, stomatitis, poor wound healing, nephrotoxicity and immunosuppression [338, 339]. These limit its application to abate neurodegeneration. Moreover, rapamycin can be toxic to mitochondrial respiration and biogenesis via the disruption of peroxisome proliferator-activated receptor gamma coactivator 1 (PGC-1) [25, 26, 91]. Finally, long-term use can produce insulin resistance, including inhibition of mTORC2 function and Akt phosphorylation as well as reductions in IRS-2 levels and glucose uptake [340]. These predict an exacerbation of T2DM [78, 341].

Formulating rapamycin or rapalogs for preferential brain delivery may overcome these systemic objections, as exampled by experimental intracerebral infusions [269, 342]. More practical strategies are being developed. So far, systemically administered nanoparticles, micelle, exosome and nanoemulsion-based rapamycin delivery systems, seeking to advantage the increased BBB permeability in AD and other neurodegenerations, is proving a challenge [343, 344]. Trials of intranasal (IN) delivery of insulin for AD has inspired a study on IN rapamycin in a mouse model of Down syndrome, finding that radial arm maze and object recognition cognitive dysfunctions are rescued along with positive effects on measures of autophagy and tau phosphorylation [345]. Small molecule catalytic mTOR inhibitors that compete with ATP have also been successfully used in Huntington models as an alternative to the allosteric rapalogs [299, 346]. Selective mTORC1 catalytic inhibitors, sparing C2, might be favored in neurodegeneration and some have been identified [347]. Alternatively, if the goal is to abate the concurrent overactivation of both Akt and mTORC1/2, dual PI3K/mTOR ATP analogues (as are in current cancer trials) could be tested. Additional approaches to selective C1 inhibition include a small molecule inhibitor of Rheb, NR1 [280].

Rapalogs such as temsirolimus and everolimus and second generation mTOR inhibitors represent major improvements in tolerance [347, 348] and hold promise as therapies against aging and AD [5]. Temsirolimus restores spatial learning and memory in 5-month-old double mutant AD transgenic mice, associated with autophagic clearance of Aβ and anti-apoptosis [349, 350]. Similar results in p-tau clearance and memory are reported in a mutant tauP301S model [214]. Intrathecal everolimus inhibited central mTOR and restored cognitive function in 3X AD mice [351].

Other drugs, affecting mTOR, are being investigated for therapy in AD. For instance, metformin, which activates AMPK (indirectly suppressing mTOR) and may also directly suppress Raptor/mTOR, can stimulate autophagy like rapamycin. Although there is some concerning epidemiological evidence pointing to an increase in AD risk in those treated with metformin [352], one clinical trial concluded that it mitigated cognitive dysfunction in MCI/AD [353]. Several *in vitro* and *in vivo* AD models also report conflicting results with metformin, either promoting amyloid aggregation and memory dysfunction or rescuing synaptic plasticity and preventing neuropathological changes. The agent cilostazol increases AMPK expression (in a Sirt-1-dependent manner), suppresses mTOR activation, increases autophagy markers beclin-1, Atg and LC3-II and promotes autophagic clearance of Aβ in N2A neurons [354]. Direct Sirt-1 overexpression, by inhibiting mTOR, promotes neurite outgrowth and cell survival during Aβ exposure [118]. Caloric restriction also stimulates AMPK, activating autophagy and preventing AD pathology in triple transgenic mice [355]. Dietary curcumin and resveratrol either reduce mTOR levels to disrupt the C1 complex or inhibit mTORC1 by activating AMPK, thereby inducing autophagy and rescuing cognitive impairment in 2X AD transgenic mice [356]. mTOR independent stimulation of autophagy, with Trehalose, is another alternative to the above approaches [357]. Several additional novel compounds that modulate mTOR and autophagy for treatment of neurodegeneration are presented in a recent review [88].

## Conclusion

Autonomous overactivation of the Akt/mTOR axis and upregulation of mTOR activity targets has been noted in several transgenic models and in AD brain (Table 1 and Fig. 3). β-amyloid is partially responsible through mechanisms including inactivation of PTEN (disinhibiting PI3K), degrading functional IRS-1 levels, and activation of mTOR. These amyloid-driven mechanisms result in a state of relative insulin/IGF resistance (inhibited homeostatic Akt activation) [358] and oxidative stress [359]. Tau phosphorylation is also driven by Akt/mTOR hyperactivation. Several interventions may break this chain of pathogenesis. For example, either genetic suppression of Aβ production or passive anti-Aβ immunization in an AD mouse model will reverse the hyperactivation of mTOR and improve cognition [162]. Similarly, genetic reduction of mTOR by one copy in transgenic 2576 AD mice is sufficient to improve central insulin signaling and cognition [160, 161].

Although some manifestation of mTOR dysregulation is unquestionably present in AD brain, and for that matter in numerous tissues of individuals with T2DM [360, 361], a definitive accounting of the exact nature and sequence of mTOR axis dysregulation is elusive. Part of this uncertainty comes from studies that have found no change or even reduced mTOR activation and/or activity parameters in AD brain and various transgenic models. Aside from the use of widely differing models, contradictions can arise from variances in disease duration and severity as well as confounding changes to mTOR-regulating and signal crosstalk proteins. Nevertheless, most all *in vivo* and *in vitro* models of AD recommend a rapamycin-like strategy. Furthermore, manipulation of mTOR is a strong treatment strategy to pursue in PD/HD and ALS.

The overall goal of mTOR-based treatment then is to either restore activity where deficient or inhibit it when excessive, in order to re-establish basal levels, reactivity to neurotrophin stimulation and nutrient status and downstream effector homeostasis. This will probably depend on the particular neurodegenerative process and type of protein aggregation, as well as disease stage. Attempts to block mTOR activity must be kept partial, in consideration of important roles in facilitating memory formation [21, 362, 363] and tissue repair involving progenitor cells [364]. The latter relates particularly to neurodegeneration and ischemic injury [365]. The balancing act includes maintenance of insulin/Akt axis homeostasis and mTORC1-dependent protein translation. Thus, over-inhibition of mTORC1 could lead to feedback hyperactivation of Akt and unchecked tumor proliferation. The inadvertent over-activation of mTOR also has the potential of tumorigenesis (for example resulting from the loss of tumor suppressor TSC1/2 function, as in Tuberous Sclerosis), but also loss of autophagy function [366], glucose intolerance via IRS-1 feedback inhibition [47] and learning impairment [330].

If the focus however is on alleviating proteotoxicity, the goal is to stimulate autophagy and protein removal. As mentioned, most studies would recommend mTOR inhibition and autophagy induction for neuroprotection, for instance in AD [119]. Still, there are other conditions for which mTOR activation would appear therapeutically beneficial. These may include where ischemia/apoptosis or stroke is the overriding pathology [363, 367, 368] noting that mTOR has anti-apoptosis properties, or where oxidative stress is of higher concern such as in certain PD and ALS models [259, 309]. Finally, the timing of mTOR inhibitor treatments can affect mTORC1 and C2 complexes differentially [369, 370]. Thus, it is plausible that an individualized balance between mTORC1 and C2 manipulations would need to be reached for each of the proteinopathies [135, 299].

The use of rapamycin or analogs to treat AD holds promise due to its many actions to increase longevity and remove toxic proteins, but toxicity concerns persist. This leaves open the possibility to target other mTOR-dependent effectors such as p70S6K1/2 [159, 371] as well as direct therapy to more selective brain regions [5]. The balance between IRS-1 inhibition (mTORC1 directed negative feedback) and Akt responsiveness to insulin (mTORC2/Rictor directed positive feedback) should be swung to favor homeostatic insulin signaling. The compelling preclinical record reviewed above calls for clinical trials to test rapamycin or other mTOR inhibitors and/or possibly mTORC2 agonists, beginning in patients with Alzheimer's disease [372].

## Abbreviations

AD: Alzheimer’s disease
Akt: Protein kinase B
ALS: Amyotrophic Lateral Sclerosis
AMPK: AMP-activated protein kinase
Atg: Autophagy-related gene/protein
BCAA: Branch-chain amino acids
BDNF: Brain-derived neurotrophic factor
DJ1 (PARK7): Deptor, domain-containing mTOR-interacting protein
4E-BP1: 4E-binding protein-1
FTD: Frontotemporal dementia
GSK-3β: Glycogen synthase kinase-3β
HD: Huntington's disease
HIF1-α: Hypoxia-inducible factor 1-α
IGF: Insulin-like growth factor
IRS-1: Insulin receptor substrate 1
LTD: Long-term depression
LTP: Long-term potentiation
MCI: Mild cognitive impairment
mLST8: Mammalian lethal with SEC13 protein 8
mSIN1: Mammalian stress-activated map kinase-interacting protein 1
mTOR: Mechanistic target of rapamycin
NO: Nitric oxide
NOS: Nitric oxide synthase
PARK7: Parkinson’s disease protein 7
PD: Parkinson’s disease
PDK1/2: Phosphoinositide-dependent kinase 1/2
PI3K: Phosphoinositide 3-kinase
PP2A: Protein phosphatase 2A
PRAS40: Proline-rich Akt substrate of 40kDa
p-tau: Phospho-tau
Protor: Protein observed with Rictor
PTEN: Phosphatase and tensin homolog
p70S6K1: p70 ribosomal S6 protein kinase 1
Rags: Ras-related GTP binding proteins
Raptor: Regulatory-associated protein of mTOR
Rheb: Ras homolog enriched in brain protein
Rictor: Rapamycin-insensitive companion of mTOR
SGK-1: Serum and glucocorticoid-regulated kinase 1
SIRT1: Sirtuin 1
TSC1/2: Tuberous sclerosis protein-complex
T2DM: Type II diabetes mellitus
ULK1: Unc-51-like kinase 1
Vps: Vacuolar protein sorting-associated protein

## References

[1] De Felice FG, Ferreira ST (2014). Inflammation, defective insulin signaling, and mitochondrial dysfunction as common molecular denominators connecting type 2 diabetes to Alzheimer disease. Diabetes.

[2] Moreira PI, Santos MS, Seica R, Oliveira CR (2007). Brain mitochondrial dysfunction as a link between Alzheimer's disease and diabetes. J Neurol Sci.

[3] Butterfield DA, Di Domenico F, Barone E (2014). Elevated risk of type 2 diabetes for development of Alzheimer disease: a key role for oxidative stress in brain. Biochim Biophys Acta.

[4] Saxton RA, Sabatini DM (2017). mTOR Signaling in Growth, Metabolism, and Disease. Cell.

[5] Richardson A, Galvan V, Lin AL, Oddo S (2015). How longevity research can lead to therapies for Alzheimer's disease: The rapamycin story. Exp Gerontol.

[6] Hoeffer CA, Klann E (2010). mTOR signaling: at the crossroads of plasticity, memory and disease. Trends Neurosci.

[7] Tang SJ, Reis G, Kang H, Gingras AC, Sonenberg N, Schuman EM (2002). A rapamycin-sensitive signaling pathway contributes to long-term synaptic plasticity in the hippocampus. Proc Natl Acad Sci U S A.

[8] Dash PK, Orsi SA, Moore AN (2006). Spatial memory formation and memory-enhancing effect of glucose involves activation of the tuberous sclerosis complex-Mammalian target of rapamycin pathway. J Neurosci.

[9] Costa-Mattioli M, Sossin WS, Klann E, Sonenberg N (2009). Translational control of long-lasting synaptic plasticity and memory. Neuron.

[10] Sutton MA, Schuman EM (2006). Dendritic protein synthesis, synaptic plasticity, and memory. Cell.

[11] Henry FE, Hockeimer W, Chen A, Mysore SP, Sutton MA (2017). Mechanistic target of rapamycin is necessary for changes in dendritic spine morphology associated with long-term potentiation. Mol Brain.

[12] Blagosklonny MV (2010). Calorie restriction: decelerating mTOR-driven aging from cells to organisms (including humans). Cell Cycle.

[13] Harrison DE, Strong R, Sharp ZD, Nelson JF, Astle CM, Flurkey K, Nadon NL, Wilkinson JE, Frenkel K, Carter CS (2009). Rapamycin fed late in life extends lifespan in genetically heterogeneous mice. Nature.

[14] Lamming DW, Ye L, Sabatini DM, Baur JA (2013). Rapalogs and mTOR inhibitors as anti-aging therapeutics. J Clin Invest.

[15] Zhang Y, Bokov A, Gelfond J, Soto V, Ikeno Y, Hubbard G, Diaz V, Sloane L, Maslin K, Treaster S (2014). Rapamycin extends life and health in C57BL/6 mice. J Gerontol A Biol Sci Med Sci.

[16] Colman RJ, Anderson RM, Johnson SC, Kastman EK, Kosmatka KJ, Beasley TM, Allison DB, Cruzen C, Simmons HA, Kemnitz JW, Weindruch R (2009). Caloric restriction delays disease onset and mortality in rhesus monkeys. Science.

[17] Swiech L, Perycz M, Malik A, Jaworski J (2008). Role of mTOR in physiology and pathology of the nervous system. Biochim Biophys Acta.

[18] Ebert DH, Greenberg ME (2013). Activity-dependent neuronal signalling and autism spectrum disorder. Nature.

[19] Santini E, Huynh TN, Klann E (2014). Mechanisms of translation control underlying long-lasting synaptic plasticity and the consolidation of long-term memory. Prog Mol Biol Transl Sci.

[20] Slipczuk L, Bekinschtein P, Katche C, Cammarota M, Izquierdo I, Medina JH (2009). BDNF activates mTOR to regulate GluR1 expression required for memory formation. PLoS One.

[21] Ramanan VK, Nho K, Shen L, Risacher SL, Kim S, McDonald BC, Farlow MR, Foroud TM, Gao S, Soininen H (2015). FASTKD2 is associated with memory and hippocampal structure in older adults. Mol Psychiatry.

[22] Stoica L, Zhu PJ, Huang W, Zhou H, Kozma SC, Costa-Mattioli M (2011). Selective pharmacogenetic inhibition of mammalian target of Rapamycin complex I (mTORC1) blocks long-term synaptic plasticity and memory storage. Proc Natl Acad Sci U S A.

[23] Bentzinger CF, Romanino K, Cloetta D, Lin S, Mascarenhas JB, Oliveri F, Xia J, Casanova E, Costa CF, Brink M (2008). Skeletal muscle-specific ablation of raptor, but not of rictor, causes metabolic changes and results in muscle dystrophy. Cell Metab.

[24] Risson V, Mazelin L, Roceri M, Sanchez H, Moncollin V, Corneloup C, Richard-Bulteau H, Vignaud A, Baas D, Defour A (2009). Muscle inactivation of mTOR causes metabolic and dystrophin defects leading to severe myopathy. J Cell Biol.

[25] Ramanathan A, Schreiber SL (2009). Direct control of mitochondrial function by mTOR. Proc Natl Acad Sci U S A.

[26] Schieke SM, Phillips D, McCoy JP, Aponte AM, Shen RF, Balaban RS, Finkel T (2006). The mammalian target of rapamycin (mTOR) pathway regulates mitochondrial oxygen consumption and oxidative capacity. J Biol Chem.

[27] Nave BT, Ouwens M, Withers DJ, Alessi DR, Shepherd PR (1999). Mammalian target of rapamycin is a direct target for protein kinase B: identification of a convergence point for opposing effects of insulin and amino-acid deficiency on protein translation. Biochem J.

[28] Hers I, Vincent EE, Tavare JM (2011). Akt signalling in health and disease. Cell Signal.

[29] Menon S, Dibble CC, Talbott G, Hoxhaj G, Valvezan AJ, Takahashi H, Cantley LC, Manning BD (2014). Spatial control of the TSC complex integrates insulin and nutrient regulation of mTORC1 at the lysosome. Cell.

[30] Noda T, Ohsumi Y (1998). Tor, a phosphatidylinositol kinase homologue, controls autophagy in yeast. J Biol Chem.

[31] Steinberg GR, Kemp BE (2009). AMPK in Health and Disease. Physiol Rev.

[32] Viollet B, Horman S, Leclerc J, Lantier L, Foretz M, Billaud M, Giri S, Andreelli F (2010). AMPK inhibition in health and disease. Crit Rev Biochem Mol Biol.

[33] Gwinn DM, Shackelford DB, Egan DF, Mihaylova MM, Mery A, Vasquez DS, Turk BE, Shaw RJ (2008). AMPK phosphorylation of raptor mediates a metabolic checkpoint. Mol Cell.

[34] Inoki K, Zhu T, Guan KL (2003). TSC2 mediates cellular energy response to control cell growth and survival. Cell.

[35] Inoki K, Li Y, Zhu T, Wu J, Guan KL (2002). TSC2 is phosphorylated and inhibited by Akt and suppresses mTOR signalling. Nat Cell Biol.

[36] Hahn-Windgassen A, Nogueira V, Chen CC, Skeen JE, Sonenberg N, Hay N (2005). Akt activates the mammalian target of rapamycin by regulating cellular ATP level and AMPK activity. J Biol Chem.

[37] Malik AR, Urbanska M, Macias M, Skalecka A, Jaworski J (2013). Beyond control of protein translation: what we have learned about the non-canonical regulation and function of mammalian target of rapamycin (mTOR). Biochim Biophys Acta.

[38] Kim J, Kundu M, Viollet B, Guan KL (2011). AMPK and mTOR regulate autophagy through direct phosphorylation of Ulk1. Nat Cell Biol.

[39] Browne GJ, Finn SG, Proud CG (2004). Stimulation of the AMP-activated protein kinase leads to activation of eukaryotic elongation factor 2 kinase and to its phosphorylation at a novel site, serine 398. J Biol Chem.

[40] Horman S, Browne G, Krause U, Patel J, Vertommen D, Bertrand L, Lavoinne A, Hue L, Proud C, Rider M (2002). Activation of AMP-activated protein kinase leads to the phosphorylation of elongation factor 2 and an inhibition of protein synthesis. Curr Biol.

[41] Kwon B, Querfurth HW (2015). Palmitate activates mTOR/p70S6K through AMPK inhibition and hypophosphorylation of raptor in skeletal muscle cells: Reversal by oleate is similar to metformin. Biochimie.

[42] Hay N, Sonenberg N (2004). Upstream and downstream of mTOR. Genes Dev.

[43] Mordier S, Iynedjian PB (2007). Activation of mammalian target of rapamycin complex 1 and insulin resistance induced by palmitate in hepatocytes. Biochem Biophys Res Commun.

[44] Ogata M, Hino S, Saito A, Morikawa K, Kondo S, Kanemoto S, Murakami T, Taniguchi M, Tanii I, Yoshinaga K (2006). Autophagy is activated for cell survival after endoplasmic reticulum stress. Mol Cell Biol.

[45] Ravikumar B, Berger Z, Vacher C, O'Kane CJ, Rubinsztein DC (2006). Rapamycin pre-treatment protects against apoptosis. Hum Mol Genet.

[46] Salvado L, Coll T, Gomez-Foix AM, Salmeron E, Barroso E, Palomer X, Vazquez-Carrera M (2013). Oleate prevents saturated-fatty-acid-induced ER stress, inflammation and insulin resistance in skeletal muscle cells through an AMPK-dependent mechanism. Diabetologia.

[47] Harrington LS, Findlay GM, Gray A, Tolkacheva T, Wigfield S, Rebholz H, Barnett J, Leslie NR, Cheng S, Shepherd PR (2004). The TSC1-2 tumor suppressor controls insulin-PI3K signaling via regulation of IRS proteins. J Cell Biol.

[48] Harrington LS, Findlay GM, Lamb RF (2005). Restraining PI3K: mTOR signalling goes back to the membrane. Trends Biochem Sci.

[49] Tzatsos A, Kandror KV (2006). Nutrients suppress phosphatidylinositol 3-kinase/Akt signaling via raptor-dependent mTOR-mediated insulin receptor substrate 1 phosphorylation. Mol Cell Biol.

[50] Perluigi M, Pupo G, Tramutola A, Cini C, Coccia R, Barone E, Head E, Butterfield DA, Di Domenico F (2014). Neuropathological role of PI3K/Akt/mTOR axis in Down syndrome brain. Biochim Biophys Acta.

[51] Tramutola A, Triplett JC, Di Domenico F, Niedowicz DM, Murphy MP, Coccia R, Perluigi M, Butterfield DA (2015). Alteration of mTOR signaling occurs early in the progression of Alzheimer disease (AD): analysis of brain from subjects with pre-clinical AD, amnestic mild cognitive impairment and late-stage AD. J Neurochem.

[52] Jewell JL, Guan KL (2013). Nutrient signaling to mTOR and cell growth. Trends Biochem Sci.

[53] Tato I, Bartrons R, Ventura F, Rosa JL (2011). Amino acids activate mammalian target of rapamycin complex 2 (mTORC2) via PI3K/Akt signaling. J Biol Chem.

[54] Kimball SR, Jefferson LS (2004). Molecular mechanisms through which amino acids mediate signaling through the mammalian target of rapamycin. Curr Opin Clin Nutr Metab Care.

[55] Reynolds TH, Bodine SC, Lawrence JC (2002). Control of Ser2448 phosphorylation in the mammalian target of rapamycin by insulin and skeletal muscle load. J Biol Chem.

[56] Dillon EL (2013). Nutritionally essential amino acids and metabolic signaling in aging. Amino Acids.

[57] Drummond MJ, Rasmussen BB (2008). Leucine-enriched nutrients and the regulation of mammalian target of rapamycin signalling and human skeletal muscle protein synthesis. Curr Opin Clin Nutr Metab Care.

[58] Liu Z, Jahn LA, Wei L, Long W, Barrett EJ (2002). Amino acids stimulate translation initiation and protein synthesis through an Akt-independent pathway in human skeletal muscle. J Clin Endocrinol Metab.

[59] Dodd KM, Tee AR (2012). Leucine and mTORC1: a complex relationship. Am J Physiol Endocrinol Metab.

[60] Kimball SR, Shantz LM, Horetsky RL, Jefferson LS (1999). Leucine regulates translation of specific mRNAs in L6 myoblasts through mTOR-mediated changes in availability of eIF4E and phosphorylation of ribosomal protein S6. J Biol Chem.

[61] Bar-Peled L, Sabatini DM (2014). Regulation of mTORC1 by amino acids. Trends Cell Biol.

[62] Ham DJ, Lynch GS, Koopman R (2016). Amino acid sensing and activation of mechanistic target of rapamycin complex 1: implications for skeletal muscle. Curr Opin Clin Nutr Metab Care.

[63] Shimobayashi M, Hall MN (2016). Multiple amino acid sensing inputs to mTORC1. Cell Res.

[64] Senturk M, Lin G, Zuo Z, Mao D, Watson E, Mikos AG, Bellen HJ (2019). Ubiquilins regulate autophagic flux through mTOR signalling and lysosomal acidification. Nat Cell Biol.

[65] Badoud F, Lam KP, DiBattista A, Perreault M, Zulyniak MA, Cattrysse B, Stephenson S, Britz-McKibbin P, Mutch DM (2014). Serum and adipose tissue amino acid homeostasis in the metabolically healthy obese. J Proteome Res.

[66] Newgard CB, An J, Bain JR, Muehlbauer MJ, Stevens RD, Lien LF, Haqq AM, Shah SH, Arlotto M, Slentz CA (2009). A branched-chain amino acid-related metabolic signature that differentiates obese and lean humans and contributes to insulin resistance. Cell Metab.

[67] Lynch CJ, Adams SH (2014). Branched-chain amino acids in metabolic signalling and insulin resistance. Nat Rev Endocrinol.

[68] Yoon MS (2016). The Emerging Role of Branched-Chain Amino Acids in Insulin Resistance and Metabolism. Nutrients.

[69] Ma L, Dong W, Wang R, Li Y, Xu B, Zhang J, Zhao Z, Wang Y (2015). Effect of caloric restriction on the SIRT1/mTOR signaling pathways in senile mice. Brain Res Bull.

[70] Ghosh HS, McBurney M, Robbins PD (2010). SIRT1 negatively regulates the mammalian target of rapamycin. PLoS One.

[71] Huang J, Dibble CC, Matsuzaki M, Manning BD (2008). The TSC1-TSC2 complex is required for proper activation of mTOR complex 2. Mol Cell Biol.

[72] Laplante M, Sabatini DM (2012). mTOR signaling in growth control and disease. Cell.

[73] Gaubitz C, Prouteau M, Kusmider B, Loewith R (2016). TORC2 Structure and Function. Trends Biochem Sci.

[74] Kaur A, Sharma S (2017). Mammalian target of rapamycin (mTOR) as a potential therapeutic target in various diseases. Inflammopharmacology.

[75] Hresko RC, Mueckler M (2005). mTOR.RICTOR is the Ser473 kinase for Akt/protein kinase B in 3T3-L1 adipocytes. J Biol Chem.

[76] Jacinto E, Loewith R, Schmidt A, Lin S, Ruegg MA, Hall A, Hall MN (2004). Mammalian TOR complex 2 controls the actin cytoskeleton and is rapamycin insensitive. Nat Cell Biol.

[77] Sarbassov DD, Ali SM, Kim DH, Guertin DA, Latek RR, Erdjument-Bromage H, Tempst P, Sabatini DM (2004). Rictor, a novel binding partner of mTOR, defines a rapamycin-insensitive and raptor-independent pathway that regulates the cytoskeleton. Curr Biol.

[78] Sarbassov DD, Ali SM, Sengupta S, Sheen JH, Hsu PP, Bagley AF, Markhard AL, Sabatini DM (2006). Prolonged rapamycin treatment inhibits mTORC2 assembly and Akt/PKB. Mol Cell.

[79] Sarbassov DD, Guertin DA, Ali SM, Sabatini DM (2005). Phosphorylation and regulation of Akt/PKB by the rictor-mTOR complex. Science.

[80] Chan TO, Tsichlis PN (2001). PDK2: a complex tail in one Akt. Sci STKE.

[81] Zhu Z, Yang C, Iyaswamy A, Krishnamoorthi S, Sreenivasmurthy SG, Liu J, Wang Z, Tong BC, Song J, Lu J (2019). Balancing mTOR Signaling and Autophagy in the Treatment of Parkinson's Disease. Int J Mol Sci.

[82] Kumar A, Harris TE, Keller SR, Choi KM, Magnuson MA, Lawrence JC (2008). Muscle-specific deletion of rictor impairs insulin-stimulated glucose transport and enhances Basal glycogen synthase activity. Mol Cell Biol.

[83] Cybulski N, Hall MN (2009). TOR complex 2: a signaling pathway of its own. Trends Biochem Sci.

[84] Sparks CA, Guertin DA (2010). Targeting mTOR: prospects for mTOR complex 2 inhibitors in cancer therapy. Oncogene.

[85] Thomanetz V, Angliker N, Cloetta D, Lustenberger RM, Schweighauser M, Oliveri F, Suzuki N, Ruegg MA (2013). Ablation of the mTORC2 component rictor in brain or Purkinje cells affects size and neuron morphology. J Cell Biol.

[86] Julien LA, Carriere A, Moreau J, Roux PP (2010). mTORC1-activated S6K1 phosphorylates Rictor on threonine 1135 and regulates mTORC2 signaling. Mol Cell Biol.

[87] Xie J, Proud CG (2014). Signaling crosstalk between the mTOR complexes. Translation (Austin).

[88] Heras-Sandoval D, Perez-Rojas JM, Pedraza-Chaverri J (2020). Novel compounds for the modulation of mTOR and autophagy to treat neurodegenerative diseases. Cell Signal.

[89] Hughes KJ, Kennedy BK (2012). Cell biology. Rapamycin paradox resolved. Science.

[90] Lamming DW, Ye L, Katajisto P, Goncalves MD, Saitoh M, Stevens DM, Davis JG, Salmon AB, Richardson A, Ahima RS (2012). Rapamycin-induced insulin resistance is mediated by mTORC2 loss and uncoupled from longevity. Science.

[91] Ye L, Varamini B, Lamming DW, Sabatini DM, Baur JA (2012). Rapamycin has a biphasic effect on insulin sensitivity in C2C12 myotubes due to sequential disruption of mTORC1 and mTORC2. Front Genet.

[92] Kim SJ, DeStefano MA, Oh WJ, Wu CC, Vega-Cotto NM, Finlan M, Liu D, Su B, Jacinto E (2012). mTOR complex 2 regulates proper turnover of insulin receptor substrate-1 via the ubiquitin ligase subunit Fbw8. Mol Cell.

[93] Stanfel MN, Shamieh LS, Kaeberlein M, Kennedy BK (2009). The TOR pathway comes of age. Biochim Biophys Acta.

[94] Hou L, Klann E (2004). Activation of the phosphoinositide 3-kinase-Akt-mammalian target of rapamycin signaling pathway is required for metabotropic glutamate receptor-dependent long-term depression. J Neurosci.

[95] Parsons RG, Gafford GM, Helmstetter FJ (2006). Translational control via the mammalian target of rapamycin pathway is critical for the formation and stability of long-term fear memory in amygdala neurons. J Neurosci.

[96] Gong R, Park CS, Abbassi NR, Tang SJ (2006). Roles of glutamate receptors and the mammalian target of rapamycin (mTOR) signaling pathway in activity-dependent dendritic protein synthesis in hippocampal neurons. J Biol Chem.

[97] Menzies FM, Fleming A, Caricasole A, Bento CF, Andrews SP, Ashkenazi A, Fullgrabe J, Jackson A, Jimenez Sanchez M, Karabiyik C (2017). Autophagy and Neurodegeneration: Pathogenic Mechanisms and Therapeutic Opportunities. Neuron.

[98] Jung CH, Jun CB, Ro SH, Kim YM, Otto NM, Cao J, Kundu M, Kim DH (2009). ULK-Atg13-FIP200 complexes mediate mTOR signaling to the autophagy machinery. Mol Biol Cell.

[99] Russell RC, Tian Y, Yuan H, Park HW, Chang YY, Kim J, Kim H, Neufeld TP, Dillin A, Guan KL (2013). ULK1 induces autophagy by phosphorylating Beclin-1 and activating VPS34 lipid kinase. Nat Cell Biol.

[100] Kim HJ, Magrane J (2011). Isolation and culture of neurons and astrocytes from the mouse brain cortex. Methods Mol Biol.

[101] Fujita N, Itoh T, Omori H, Fukuda M, Noda T, Yoshimori T (2008). The Atg16L complex specifies the site of LC3 lipidation for membrane biogenesis in autophagy. Mol Biol Cell.

[102] Kaushik S, Cuervo AM (2018). The coming of age of chaperone-mediated autophagy. Nat Rev Mol Cell Biol.

[103] Hara T, Nakamura K, Matsui M, Yamamoto A, Nakahara Y, Suzuki-Migishima R, Yokoyama M, Mishima K, Saito I, Okano H, Mizushima N (2006). Suppression of basal autophagy in neural cells causes neurodegenerative disease in mice. Nature.

[104] Komatsu M, Waguri S, Chiba T, Murata S, Iwata J, Tanida I, Ueno T, Koike M, Uchiyama Y, Kominami E, Tanaka K (2006). Loss of autophagy in the central nervous system causes neurodegeneration in mice. Nature.

[105] Goodman CA, Mayhew DL, Hornberger TA (2011). Recent progress toward understanding the molecular mechanisms that regulate skeletal muscle mass. Cell Signal.

[106] Kou X, Chen D, Chen N (2019). Physical Activity Alleviates Cognitive Dysfunction of Alzheimer's Disease through Regulating the mTOR Signaling Pathway. Int J Mol Sci.

[107] Smith ED, Prieto GA, Tong L, Sears-Kraxberger I, Rice JD, Steward O, Cotman CW (2014). Rapamycin and interleukin-1beta impair brain-derived neurotrophic factor-dependent neuron survival by modulating autophagy. J Biol Chem.

[108] Tian Y, Bustos V, Flajolet M, Greengard P (2011). A small-molecule enhancer of autophagy decreases levels of Abeta and APP-CTF via Atg5-dependent autophagy pathway. FASEB J.

[109] Wu T, Wang MC, Jing L, Liu ZY, Guo H, Liu Y, Bai YY, Cheng YZ, Nan KJ, Liang X (2015). Autophagy facilitates lung adenocarcinoma resistance to cisplatin treatment by activation of AMPK/mTOR signaling pathway. Drug Des Devel Ther.

[110] Vingtdeux V, Chandakkar P, Zhao H, d'Abramo C, Davies P, Marambaud P (2011). Novel synthetic small-molecule activators of AMPK as enhancers of autophagy and amyloid-beta peptide degradation. FASEB J.

[111] Plaza-Zabala A, Sierra-Torre V, Sierra A (2017). Autophagy and Microglia: Novel Partners in Neurodegeneration and Aging. Int J Mol Sci.

[112] Li Q, Liu Y, Sun M (2017). Autophagy and Alzheimer's Disease. Cell Mol Neurobiol.

[113] Trigiani LJ, Hamel E (2017). An endothelial link between the benefits of physical exercise in dementia. J Cereb Blood Flow Metab.

[114] Braak H, Braak E (1991). Neuropathological stageing of Alzheimer-related changes. Acta Neuropathol.

[115] Borlikova GG, Trejo M, Mably AJ, Mc Donald JM, Sala Frigerio C, Regan CM, Murphy KJ, Masliah E, Walsh DM (2013). Alzheimer brain-derived amyloid beta-protein impairs synaptic remodeling and memory consolidation. Neurobiol Aging.

[116] Douglas PM, Dillin A (2010). Protein homeostasis and aging in neurodegeneration. J Cell Biol.

[117] Cohen E, Paulsson JF, Blinder P, Burstyn-Cohen T, Du D, Estepa G, Adame A, Pham HM, Holzenberger M, Kelly JW (2009). Reduced IGF-1 signaling delays age-associated proteotoxicity in mice. Cell.

[118] Guo W, Qian L, Zhang J, Zhang W, Morrison A, Hayes P, Wilson S, Chen T, Zhao J (2011). Sirt1 overexpression in neurons promotes neurite outgrowth and cell survival through inhibition of the mTOR signaling. J Neurosci Res.

[119] Cai Z, Yan LJ (2013). Rapamycin, Autophagy, and Alzheimer's Disease. J Biochem Pharmacol Res.

[120] Roberts MN, Wallace MA, Tomilov AA, Zhou Z, Marcotte GR, Tran D, Perez G, Gutierrez-Casado E, Koike S, Knotts TA (2017). A Ketogenic Diet Extends Longevity and Healthspan in Adult Mice. Cell Metab.

[121] Carro E, Trejo JL, Spuch C, Bohl D, Heard JM, Torres-Aleman I (2006). Blockade of the insulin-like growth factor I receptor in the choroid plexus originates Alzheimer's-like neuropathology in rodents: new cues into the human disease?. Neurobiol Aging.

[122] Watson GS, Craft S (2003). The role of insulin resistance in the pathogenesis of Alzheimer's disease: implications for treatment. CNS Drugs.

[123] Ott A, Stolk RP, van Harskamp F, Pols HA, Hofman A, Breteler MM (1999). Diabetes mellitus and the risk of dementia: The Rotterdam Study. Neurology.

[124] Biessels GJ, Staekenborg S, Brunner E, Brayne C, Scheltens P (2006). Risk of dementia in diabetes mellitus: a systematic review. Lancet Neurol.

[125] Luchsinger JA, Patel B, Tang MX, Schupf N, Mayeux R (2007). Measures of adiposity and dementia risk in elderly persons. Arch Neurol.

[126] Schrijvers EM, Witteman JC, Sijbrands EJ, Hofman A, Koudstaal PJ, Breteler MM (2010). Insulin metabolism and the risk of Alzheimer disease: the Rotterdam Study. Neurology.

[127] Ho L, Qin W, Pompl PN, Xiang Z, Wang J, Zhao Z, Peng Y, Cambareri G, Rocher A, Mobbs CV (2004). Diet-induced insulin resistance promotes amyloidosis in a transgenic mouse model of Alzheimer's disease. FASEB J.

[128] Zhao L, Teter B, Morihara T, Lim GP, Ambegaokar SS, Ubeda OJ, Frautschy SA, Cole GM (2004). Insulin-degrading enzyme as a downstream target of insulin receptor signaling cascade: implications for Alzheimer's disease intervention. J Neurosci.

[129] Rivera EJ, Goldin A, Fulmer N, Tavares R, Wands JR, de la Monte SM (2005). Insulin and insulin-like growth factor expression and function deteriorate with progression of Alzheimer's disease: link to brain reductions in acetylcholine. J Alzheimers Dis.

[130] Steen E, Terry BM, Rivera EJ, Cannon JL, Neely TR, Tavares R, Xu XJ, Wands JR, de la Monte SM (2005). Impaired insulin and insulin-like growth factor expression and signaling mechanisms in Alzheimer's disease--is this type 3 diabetes?. J Alzheimers Dis.

[131] de la Monte SM, Wands JR (2008). Alzheimer's disease is type 3 diabetes-evidence reviewed. J Diabetes Sci Technol.

[132] Correia SC, Santos RX, Perry G, Zhu X, Moreira PI, Smith MA (2011). Insulin-resistant brain state: the culprit in sporadic Alzheimer's disease?. Ageing Res Rev.

[133] de la Monte SM (2017). Insulin Resistance and Neurodegeneration: Progress Towards the Development of New Therapeutics for Alzheimer's Disease. Drugs.

[134] Diehl T, Mullins R, Kapogiannis D (2017). Insulin resistance in Alzheimer's disease. Transl Res.

[135] Lee HK, Kwon B, Lemere CA, de la Monte S, Itamura K, Ha AY, Querfurth HW (2017). mTORC2 (Rictor) in Alzheimer's Disease and Reversal of Amyloid-beta Expression-Induced Insulin Resistance and Toxicity in Rat Primary Cortical Neurons. J Alzheimers Dis.

[136] Talbot K, Wang HY, Kazi H, Han LY, Bakshi KP, Stucky A, Fuino RL, Kawaguchi KR, Samoyedny AJ, Wilson RS (2012). Demonstrated brain insulin resistance in Alzheimer's disease patients is associated with IGF-1 resistance, IRS-1 dysregulation, and cognitive decline. J Clin Invest.

[137] O'Neill C (2013). PI3-kinase/Akt/mTOR signaling: impaired on/off switches in aging, cognitive decline and Alzheimer's disease. Exp Gerontol.

[138] Zhao WQ, De Felice FG, Fernandez S, Chen H, Lambert MP, Quon MJ, Krafft GA, Klein WL (2008). Amyloid beta oligomers induce impairment of neuronal insulin receptors. FASEB J.

[139] Lourenco MV, Clarke JR, Frozza RL, Bomfim TR, Forny-Germano L, Batista AF, Sathler LB, Brito-Moreira J, Amaral OB, Silva CA (2013). TNF-alpha mediates PKR-dependent memory impairment and brain IRS-1 inhibition induced by Alzheimer's beta-amyloid oligomers in mice and monkeys. Cell Metab.

[140] Norwitz NG, Mota AS, Norwitz SG, Clarke K (2019). Multi-Loop Model of Alzheimer Disease: An Integrated Perspective on the Wnt/GSK3beta, alpha-Synuclein, and Type 3 Diabetes Hypotheses. Front Aging Neurosci.

[141] Cuesto G, Enriquez-Barreto L, Carames C, Cantarero M, Gasull X, Sandi C, Ferrus A, Acebes A, Morales M (2011). Phosphoinositide-3-kinase activation controls synaptogenesis and spinogenesis in hippocampal neurons. J Neurosci.

[142] Yi JH, Baek SJ, Heo S, Park HJ, Kwon H, Lee S, Jung J, Park SJ, Kim BC, Lee YC (2018). Direct pharmacological Akt activation rescues Alzheimer's disease like memory impairments and aberrant synaptic plasticity. Neuropharmacology.

[143] Talboom JS, Velazquez R, Oddo S (2015). The mammalian target of rapamycin at the crossroad between cognitive aging and Alzheimer's disease. NPJ Aging Mech Dis.

[144] An WL, Cowburn RF, Li L, Braak H, Alafuzoff I, Iqbal K, Iqbal IG, Winblad B, Pei JJ (2003). Up-regulation of phosphorylated/activated p70 S6 kinase and its relationship to neurofibrillary pathology in Alzheimer's disease. Am J Pathol.

[145] Griffin RJ, Moloney A, Kelliher M, Johnston JA, Ravid R, Dockery P, O'Connor R, O'Neill C (2005). Activation of Akt/PKB, increased phosphorylation of Akt substrates and loss and altered distribution of Akt and PTEN are features of Alzheimer's disease pathology. J Neurochem.

[146] Li X, Alafuzoff I, Soininen H, Winblad B, Pei JJ (2005). Levels of mTOR and its downstream targets 4E-BP1, eEF2, and eEF2 kinase in relationships with tau in Alzheimer's disease brain. FEBS J.

[147] Majd S, Power JHT (2018). Oxidative Stress and Decreased Mitochondrial Superoxide Dismutase 2 and Peroxiredoxins 1 and 4 Based Mechanism of Concurrent Activation of AMPK and mTOR in Alzheimer's Disease. Curr Alzheimer Res.

[148] Pei JJ, Bjorkdahl C, Zhang H, Zhou X, Winblad B (2008). p70 S6 kinase and tau in Alzheimer's disease. J Alzheimers Dis.

[149] Pei JJ, Hugon J (2008). mTOR-dependent signalling in Alzheimer's disease. J Cell Mol Med.

[150] Pei JJ, Khatoon S, An WL, Nordlinder M, Tanaka T, Braak H, Tsujio I, Takeda M, Alafuzoff I, Winblad B (2003). Role of protein kinase B in Alzheimer's neurofibrillary pathology. Acta Neuropathol (Berl).

[151] Sonoda Y, Tooyama I, Mukai H, Maeda K, Akiyama H, Kawamata T (2016). S6 kinase phosphorylated at T229 is involved in tau and actin pathologies in Alzheimer's disease. Neuropathology.

[152] Sun YX, Ji X, Mao X, Xie L, Jia J, Galvan V, Greenberg DA, Jin K (2014). Differential activation of mTOR complex 1 signaling in human brain with mild to severe Alzheimer's disease. J Alzheimers Dis.

[153] Iyer AM, van Scheppingen J, Milenkovic I, Anink JJ, Adle-Biassette H, Kovacs GG, Aronica E (2014). mTOR Hyperactivation in down syndrome hippocampus appears early during development. J Neuropathol Exp Neurol.

[154] Bhaskar K, Miller M, Chludzinski A, Herrup K, Zagorski M, Lamb BT (2009). The PI3K-Akt-mTOR pathway regulates Abeta oligomer induced neuronal cell cycle events. Mol Neurodegener.

[155] Caccamo A, Majumder S, Richardson A, Strong R, Oddo S (2010). Molecular interplay between mammalian target of rapamycin (mTOR), amyloid-beta, and Tau: effects on cognitive impairments. J Biol Chem.

[156] Caccamo A, Maldonado MA, Majumder S, Medina DX, Holbein W, Magri A, Oddo S (2011). Naturally secreted amyloid-beta increases mammalian target of rapamycin (mTOR) activity via a PRAS40-mediated mechanism. J Biol Chem.

[157] Ma T, Hoeffer CA, Capetillo-Zarate E, Yu F, Wong H, Lin MT, Tampellini D, Klann E, Blitzer RD, Gouras GK (2010). Dysregulation of the mTOR pathway mediates impairment of synaptic plasticity in a mouse model of Alzheimer's disease. PLoS One.

[158] Pei JJ, An WL, Zhou XW, Nishimura T, Norberg J, Benedikz E, Gotz J, Winblad B (2006). P70 S6 kinase mediates tau phosphorylation and synthesis. FEBS Lett.

[159] Caccamo A, Branca C, Talboom JS, Shaw DM, Turner D, Ma L, Messina A, Huang Z, Wu J, Oddo S (2015). Reducing Ribosomal Protein S6 Kinase 1 Expression Improves Spatial Memory and Synaptic Plasticity in a Mouse Model of Alzheimer's Disease. J Neurosci.

[160] Caccamo A, De Pinto V, Messina A, Branca C, Oddo S (2014). Genetic reduction of mammalian target of rapamycin ameliorates Alzheimer's disease-like cognitive and pathological deficits by restoring hippocampal gene expression signature. J Neurosci.

[161] Caccamo A, Belfiore R, Oddo S (2018). Genetically reducing mTOR signaling rescues central insulin dysregulation in a mouse model of Alzheimer's disease. Neurobiol Aging.

[162] Chiang ACA, Fowler SW, Savjani RR, Hilsenbeck SG, Wallace CE, Cirrito JR, Das P, Jankowsky JL (2018). Combination anti-Abeta treatment maximizes cognitive recovery and rebalances mTOR signaling in APP mice. J Exp Med.

[163] Ou Z, Kong X, Sun X, He X, Zhang L, Gong Z, Huang J, Xu B, Long D, Li J (2018). Metformin treatment prevents amyloid plaque deposition and memory impairment in APP/PS1 mice. Brain Behav Immun.

[164] Perluigi M, Di Domenico F, Butterfield DA (2015). mTOR signaling in aging and neurodegeneration: At the crossroad between metabolism dysfunction and impairment of autophagy. Neurobiol Dis.

[165] Norambuena A, Wallrabe H, McMahon L, Silva A, Swanson E, Khan SS, Baerthlein D, Kodis E, Oddo S, Mandell JW, Bloom GS (2017). mTOR and neuronal cell cycle reentry: How impaired brain insulin signaling promotes Alzheimer's disease. Alzheimers Dement.

[166] Lafay-Chebassier C, Paccalin M, Page G, Barc-Pain S, Perault-Pochat MC, Gil R, Pradier L, Hugon J (2005). mTOR/p70S6k signalling alteration by Abeta exposure as well as in APP-PS1 transgenic models and in patients with Alzheimer's disease. J Neurochem.

[167] Lafay-Chebassier C, Perault-Pochat MC, Page G, Rioux Bilan A, Damjanac M, Pain S, Houeto JL, Gil R, Hugon J (2006). The immunosuppressant rapamycin exacerbates neurotoxicity of Abeta peptide. J Neurosci Res.

[168] Ahmad F, Singh K, Das D, Gowaikar R, Shaw E, Ramachandran A, Rupanagudi KV, Kommaddi RP, Bennett DA, Ravindranath V (2017). Reactive Oxygen Species-Mediated Loss of Synaptic Akt1 Signaling Leads to Deficient Activity-Dependent Protein Translation Early in Alzheimer's Disease. Antioxid Redox Signal.

[169] Lee HK, Kumar P, Fu Q, Rosen KM, Querfurth HW (2009). The insulin/Akt signaling pathway is targeted by intracellular beta-amyloid. Mol Biol Cell.

[170] Francois A, Rioux Bilan A, Quellard N, Fernandez B, Janet T, Chassaing D, Paccalin M, Terro F, Page G (2014). Longitudinal follow-up of autophagy and inflammation in brain of APPswePS1dE9 transgenic mice. J Neuroinflammation.

[171] Damjanac M, Rioux Bilan A, Paccalin M, Pontcharraud R, Fauconneau B, Hugon J, Page G (2008). Dissociation of Akt/PKB and ribosomal S6 kinase signaling markers in a transgenic mouse model of Alzheimer's disease. Neurobiol Dis.

[172] Ma T, Chen Y, Vingtdeux V, Zhao H, Viollet B, Marambaud P, Klann E (2014). Inhibition of AMP-activated protein kinase signaling alleviates impairments in hippocampal synaptic plasticity induced by amyloid beta. J Neurosci.

[173] Chen TJ, Wang DC, Chen SS (2009). Amyloid-beta interrupts the PI3K-Akt-mTOR signaling pathway that could be involved in brain-derived neurotrophic factor-induced Arc expression in rat cortical neurons. J Neurosci Res.

[174] Xue Z, Guo Y, Zhang S, Huang L, He Y, Fang R, Fang Y (2014). Beta-asarone attenuates amyloid beta-induced autophagy via Akt/mTOR pathway in PC12 cells. Eur J Pharmacol.

[175] Chano T, Okabe H, Hulette CM (2007). RB1CC1 insufficiency causes neuronal atrophy through mTOR signaling alteration and involved in the pathology of Alzheimer's diseases. Brain Res.

[176] Siman R, Cocca R, Dong Y (2015). The mTOR Inhibitor Rapamycin Mitigates Perforant Pathway Neurodegeneration and Synapse Loss in a Mouse Model of Early-Stage Alzheimer-Type Tauopathy. PLoS One.

[177] Avrahami L, Farfara D, Shaham-Kol M, Vassar R, Frenkel D, Eldar-Finkelman H (2013). Inhibition of glycogen synthase kinase-3 ameliorates beta-amyloid pathology and restores lysosomal acidification and mammalian target of rapamycin activity in the Alzheimer disease mouse model: in vivo and in vitro studies. J Biol Chem.

[178] O'Neill C, Kiely AP, Coakley MF, Manning S, Long-Smith CM (2012). Insulin and IGF-1 signalling: longevity, protein homoeostasis and Alzheimer's disease. Biochem Soc Trans.

[179] Di Domenico F, Tramutola A, Foppoli C, Head E, Perluigi M, Butterfield DA (2018). mTOR in Down syndrome: Role in Ass and tau neuropathology and transition to Alzheimer disease-like dementia. Free Radic Biol Med.

[180] Cheng J, North BJ, Zhang T, Dai X, Tao K, Guo J, Wei W (2018). The emerging roles of protein homeostasis-governing pathways in Alzheimer's disease. Aging Cell.

[181] Hodges SL, Reynolds CD, Smith GD, Jefferson TS, Nolan SO, Lugo JN (2018). Molecular interplay between hyperactive mammalian target of rapamycin signaling and Alzheimer's disease neuropathology in the NS-Pten knockout mouse model. Neuroreport.

[182] Gupta A, Dey CS (2012). PTEN, a widely known negative regulator of insulin/PI3K signaling, positively regulates neuronal insulin resistance. Mol Biol Cell.

[183] Knafo S, Sanchez-Puelles C, Palomer E, Delgado I, Draffin JE, Mingo J, Wahle T, Kaleka K, Mou L, Pereda-Perez I (2016). PTEN recruitment controls synaptic and cognitive function in Alzheimer's models. Nat Neurosci.

[184] Rickle A, Bogdanovic N, Volkmann I, Zhou X, Pei JJ, Winblad B, Cowburn RF (2006). PTEN levels in Alzheimer's disease medial temporal cortex. Neurochem Int.

[185] Gandhi S, Muqit MM, Stanyer L, Healy DG, Abou-Sleiman PM, Hargreaves I, Heales S, Ganguly M, Parsons L, Lees AJ (2006). PINK1 protein in normal human brain and Parkinson's disease. Brain.

[186] Vingtdeux V, Giliberto L, Zhao H, Chandakkar P, Wu Q, Simon JE, Janle EM, Lobo J, Ferruzzi MG, Davies P, Marambaud P (2010). AMP-activated protein kinase signaling activation by resveratrol modulates amyloid-beta peptide metabolism. J Biol Chem.

[187] Lu J, Wu DM, Zheng YL, Hu B, Zhang ZF, Shan Q, Zheng ZH, Liu CM, Wang YJ (2010). Quercetin activates AMP-activated protein kinase by reducing PP2C expression protecting old mouse brain against high cholesterol-induced neurotoxicity. J Pathol.

[188] Seixas da Silva GS, Melo HM, Lourenco MV, Lyra ESNM, de Carvalho MB, Alves-Leon SV, de Souza JM, Klein WL, da-Silva WS, Ferreira ST, De Felice FG (2017). Amyloid-beta oligomers transiently inhibit AMP-activated kinase and cause metabolic defects in hippocampal neurons. J Biol Chem.

[189] Majumder S, Caccamo A, Medina DX, Benavides AD, Javors MA, Kraig E, Strong R, Richardson A, Oddo S (2012). Lifelong rapamycin administration ameliorates age-dependent cognitive deficits by reducing IL-1beta and enhancing NMDA signaling. Aging Cell.

[190] Kapogiannis D, Boxer A, Schwartz JB, Abner EL, Biragyn A, Masharani U, Frassetto L, Petersen RC, Miller BL, Goetzl EJ (2015). Dysfunctionally phosphorylated type 1 insulin receptor substrate in neural-derived blood exosomes of preclinical Alzheimer's disease. FASEB J.

[191] Zhao WQ, Townsend M (2009). Insulin resistance and amyloidogenesis as common molecular foundation for type 2 diabetes and Alzheimer's disease. Biochim Biophys Acta.

[192] Spilman P, Podlutskaya N, Hart MJ, Debnath J, Gorostiza O, Bredesen D, Richardson A, Strong R, Galvan V (2010). Inhibition of mTOR by rapamycin abolishes cognitive deficits and reduces amyloid-beta levels in a mouse model of Alzheimer's disease. PLoS One.

[193] Luchsinger JA (2008). Adiposity, hyperinsulinemia, diabetes and Alzheimer's disease: an epidemiological perspective. Eur J Pharmacol.

[194] Boland B, Kumar A, Lee S, Platt FM, Wegiel J, Yu WH, Nixon RA (2008). Autophagy induction and autophagosome clearance in neurons: relationship to autophagic pathology in Alzheimer's disease. J Neurosci.

[195] Cai Z, Yan LJ, Li K, Quazi SH, Zhao B (2012). Roles of AMP-activated protein kinase in Alzheimer's disease. NeuroMolecular Med.

[196] Pierce A, Podlutskaya N, Halloran JJ, Hussong SA, Lin PY, Burbank R, Hart MJ, Galvan V (2013). Over-expression of heat shock factor 1 phenocopies the effect of chronic inhibition of TOR by rapamycin and is sufficient to ameliorate Alzheimer's-like deficits in mice modeling the disease. J Neurochem.

[197] Son SM, Shin HJ, Byun J, Kook SY, Moon M, Chang YJ, Mook-Jung I (2016). Metformin Facilitates Amyloid-beta Generation by beta- and gamma-Secretases via Autophagy Activation. J Alzheimers Dis.

[198] Zhang S, Salemi J, Hou H, Zhu Y, Mori T, Giunta B, Obregon D, Tan J (2010). Rapamycin promotes beta-amyloid production via ADAM-10 inhibition. Biochem Biophys Res Commun.

[199] Shahani N, Pryor W, Swarnkar S, Kholodilov N, Thinakaran G, Burke RE, Subramaniam S (2014). Rheb GTPase regulates beta-secretase levels and amyloid beta generation. J Biol Chem.

[200] Sun Q, Wei LL, Zhang M, Li TX, Yang C, Deng SP, Zeng QC (2019). Rapamycin inhibits activation of AMPK-mTOR signaling pathway-induced Alzheimer's disease lesion in hippocampus of rats with type 2 diabetes mellitus. Int J Neurosci.

[201] Tang Z, Ioja E, Bereczki E, Hultenby K, Li C, Guan Z, Winblad B, Pei JJ (2015). mTor mediates tau localization and secretion: Implication for Alzheimer's disease. Biochim Biophys Acta.

[202] Caccamo A, Magri A, Medina DX, Wisely EV, Lopez-Aranda MF, Silva AJ, Oddo S (2013). mTOR regulates tau phosphorylation and degradation: implications for Alzheimer's disease and other tauopathies. Aging Cell.

[203] Kickstein E, Krauss S, Thornhill P, Rutschow D, Zeller R, Sharkey J, Williamson R, Fuchs M, Kohler A, Glossmann H (2010). Biguanide metformin acts on tau phosphorylation via mTOR/protein phosphatase 2A (PP2A) signaling. Proc Natl Acad Sci U S A.

[204] Lee MJ, Lee JH, Rubinsztein DC (2013). Tau degradation: the ubiquitin-proteasome system versus the autophagy-lysosome system. Prog Neurobiol.

[205] Li X, An WL, Alafuzoff I, Soininen H, Winblad B, Pei JJ (2004). Phosphorylated eukaryotic translation factor 4E is elevated in Alzheimer brain. Neuroreport.

[206] Oddo S (2012). The role of mTOR signaling in Alzheimer disease. Front Biosci.

[207] Liu Y, Su Y, Wang J, Sun S, Wang T, Qiao X, Run X, Li H, Liang Z (2013). Rapamycin decreases tau phosphorylation at Ser214 through regulation of cAMP-dependent kinase. Neurochem Int.

[208] Vingtdeux V, Davies P, Dickson DW, Marambaud P (2011). AMPK is abnormally activated in tangle- and pre-tangle-bearing neurons in Alzheimer's disease and other tauopathies. Acta Neuropathol.

[209] Potter WB, O'Riordan KJ, Barnett D, Osting SM, Wagoner M, Burger C, Roopra A (2010). Metabolic regulation of neuronal plasticity by the energy sensor AMPK. PLoS One.

[210] Zimmermann HR, Yang W, Kasica NP, Zhou X, Wang X, Beckelman BC, Lee J, Furdui CM, Keene CD, Ma T (2020). Brain-specific repression of AMPKalpha1 alleviates pathophysiology in Alzheimer's model mice. J Clin Invest.

[211] Wang X, Zimmermann HR, Ma T (2019). Therapeutic Potential of AMP-Activated Protein Kinase in Alzheimer's Disease. J Alzheimers Dis.

[212] Khurana V, Lu Y, Steinhilb ML, Oldham S, Shulman JM, Feany MB (2006). TOR-mediated cell-cycle activation causes neurodegeneration in a Drosophila tauopathy model. Curr Biol.

[213] Steinhilb ML, Dias-Santagata D, Fulga TA, Felch DL, Feany MB (2007). Tau phosphorylation sites work in concert to promote neurotoxicity in vivo. Mol Biol Cell.

[214] Frederick C, Ando K, Leroy K, Heraud C, Suain V, Buee L, Brion JP (2015). Rapamycin ester analog CCI-779/Temsirolimus alleviates tau pathology and improves motor deficit in mutant tau transgenic mice. J Alzheimers Dis.

[215] Nixon RA (2013). The role of autophagy in neurodegenerative disease. Nat Med.

[216] Miners JS, Baig S, Palmer J, Palmer LE, Kehoe PG, Love S (2008). Abeta-degrading enzymes in Alzheimer's disease. Brain Pathol.

[217] Son SM, Jung ES, Shin HJ, Byun J, Mook-Jung I (2012). Abeta-induced formation of autophagosomes is mediated by RAGE-CaMKKbeta-AMPK signaling. Neurobiol Aging.

[218] Das U, Scott DA, Ganguly A, Koo EH, Tang Y, Roy S (2013). Activity-induced convergence of APP and BACE-1 in acidic microdomains via an endocytosis-dependent pathway. Neuron.

[219] Yu WH, Cuervo AM, Kumar A, Peterhoff CM, Schmidt SD, Lee JH, Mohan PS, Mercken M, Farmery MR, Tjernberg LO (2005). Macroautophagy--a novel Beta-amyloid peptide-generating pathway activated in Alzheimer's disease. J Cell Biol.

[220] Majumder S, Richardson A, Strong R, Oddo S (2011). Inducing autophagy by rapamycin before, but not after, the formation of plaques and tangles ameliorates cognitive deficits. PLoS One.

[221] Congdon EE, Wu JW, Myeku N, Figueroa YH, Herman M, Marinec PS, Gestwicki JE, Dickey CA, Yu WH, Duff KE (2012). Methylthioninium chloride (methylene blue) induces autophagy and attenuates tauopathy in vitro and in vivo. Autophagy.

[222] Rodriguez-Martin T, Cuchillo-Ibanez I, Noble W, Nyenya F, Anderton BH, Hanger DP (2013). Tau phosphorylation affects its axonal transport and degradation. Neurobiol Aging.

[223] Huang W, Zhu PJ, Zhang S, Zhou H, Stoica L, Galiano M, Krnjevic K, Roman G, Costa-Mattioli M (2013). mTORC2 controls actin polymerization required for consolidation of long-term memory. Nat Neurosci.

[224] Tsokas P, Grace EA, Chan P, Ma T, Sealfon SC, Iyengar R, Landau EM, Blitzer RD (2005). Local protein synthesis mediates a rapid increase in dendritic elongation factor 1A after induction of late long-term potentiation. J Neurosci.

[225] Lee CC, Huang CC, Wu MY, Hsu KS (2005). Insulin stimulates postsynaptic density-95 protein translation via the phosphoinositide 3-kinase-Akt-mammalian target of rapamycin signaling pathway. J Biol Chem.

[226] Takei N, Inamura N, Kawamura M, Namba H, Hara K, Yonezawa K, Nawa H (2004). Brain-derived neurotrophic factor induces mammalian target of rapamycin-dependent local activation of translation machinery and protein synthesis in neuronal dendrites. J Neurosci.

[227] Auerbach BD, Osterweil EK, Bear MF (2011). Mutations causing syndromic autism define an axis of synaptic pathophysiology. Nature.

[228] Prabowo AS, Anink JJ, Lammens M, Nellist M, van den Ouweland AM, Adle-Biassette H, Sarnat HB, Flores-Sarnat L, Crino PB, Aronica E (2013). Fetal brain lesions in tuberous sclerosis complex: TORC1 activation and inflammation. Brain Pathol.

[229] Darnell JC, Jensen KB, Jin P, Brown V, Warren ST, Darnell RB (2001). Fragile X mental retardation protein targets G quartet mRNAs important for neuronal function. Cell.

[230] Martin BS, Huntsman MM (2012). Pathological plasticity in fragile X syndrome. Neural Plast.

[231] Bolduc FV, Bell K, Cox H, Broadie KS, Tully T (2008). Excess protein synthesis in Drosophila fragile X mutants impairs long-term memory. Nat Neurosci.

[232] Klann E, Dever TE (2004). Biochemical mechanisms for translational regulation in synaptic plasticity. Nat Rev Neurosci.

[233] Van Skike CE, Galvan V (2018). A Perfect sTORm: The Role of the Mammalian Target of Rapamycin (mTOR) in Cerebrovascular Dysfunction of Alzheimer's Disease: A Mini-Review. Gerontology.

[234] Lin AL, Zheng W, Halloran JJ, Burbank RR, Hussong SA, Hart MJ, Javors M, Shih YY, Muir E, Solano Fonseca R (2013). Chronic rapamycin restores brain vascular integrity and function through NO synthase activation and improves memory in symptomatic mice modeling Alzheimer's disease. J Cereb Blood Flow Metab.

[235] Perez SE, He B, Nadeem M, Wuu J, Ginsberg SD, Ikonomovic MD, Mufson EJ (2015). Hippocampal endosomal, lysosomal, and autophagic dysregulation in mild cognitive impairment: correlation with abeta and tau pathology. J Neuropathol Exp Neurol.

[236] Paccalin M, Pain-Barc S, Pluchon C, Paul C, Besson MN, Carret-Rebillat AS, Rioux-Bilan A, Gil R, Hugon J (2006). Activated mTOR and PKR kinases in lymphocytes correlate with memory and cognitive decline in Alzheimer's disease. Dement Geriatr Cogn Disord.

[237] Li L, Zhang S, Zhang X, Li T, Tang Y, Liu H, Yang W, Le W (2013). Autophagy enhancer carbamazepine alleviates memory deficits and cerebral amyloid-beta pathology in a mouse model of Alzheimer's disease. Curr Alzheimer Res.

[238] Jimenez S, Torres M, Vizuete M, Sanchez-Varo R, Sanchez-Mejias E, Trujillo-Estrada L, Carmona-Cuenca I, Caballero C, Ruano D, Gutierrez A, Vitorica J (2011). Age-dependent accumulation of soluble amyloid beta (Abeta) oligomers reverses the neuroprotective effect of soluble amyloid precursor protein-alpha (sAPP(alpha)) by modulating phosphatidylinositol 3-kinase (PI3K)/Akt-GSK-3beta pathway in Alzheimer mouse model. J Biol Chem.

[239] Morel M, Couturier J, Lafay-Chebassier C, Paccalin M, Page G (2009). PKR, the double stranded RNA-dependent protein kinase as a critical target in Alzheimer's disease. J Cell Mol Med.

[240] Ma YQ, Wu DK, Liu JK (2013). mTOR and tau phosphorylated proteins in the hippocampal tissue of rats with type 2 diabetes and Alzheimer's disease. Mol Med Rep.

[241] de la Monte SM, Tong M, Schiano I, Didsbury J (2017). Improved Brain Insulin/IGF Signaling and Reduced Neuroinflammation with T3D-959 in an Experimental Model of Sporadic Alzheimer's Disease. J Alzheimers Dis.

[242] Di Domenico F, Barone E, Perluigi M, Butterfield DA (2017). The Triangle of Death in Alzheimer's Disease Brain: The Aberrant Cross-Talk Among Energy Metabolism, Mammalian Target of Rapamycin Signaling, and Protein Homeostasis Revealed by Redox Proteomics. Antioxid Redox Signal.

[243] Thornton C, Bright NJ, Sastre M, Muckett PJ, Carling D (2011). AMP-activated protein kinase (AMPK) is a tau kinase, activated in response to amyloid beta-peptide exposure. Biochem J.

[244] Tang Z, Baykal AT, Gao H, Quezada HC, Zhang H, Bereczki E, Serhatli M, Baykal B, Acioglu C, Wang S (2014). mTor is a signaling hub in cell survival: a mass-spectrometry-based proteomics investigation. J Proteome Res.

[245] Jacinto E, Facchinetti V, Liu D, Soto N, Wei S, Jung SY, Huang Q, Qin J, Su B (2006). SIN1/MIP1 maintains rictor-mTOR complex integrity and regulates Akt phosphorylation and substrate specificity. Cell.

[246] Goncharova EA, Goncharov DA, Li H, Pimtong W, Lu S, Khavin I, Krymskaya VP (2011). mTORC2 is required for proliferation and survival of TSC2-null cells. Mol Cell Biol.

[247] Han EK, Leverson JD, McGonigal T, Shah OJ, Woods KW, Hunter T, Giranda VL, Luo Y (2007). Akt inhibitor A-443654 induces rapid Akt Ser-473 phosphorylation independent of mTORC1 inhibition. Oncogene.

[248] Abbott JJ, Howlett DR, Francis PT, Williams RJ (2008). Abeta(1-42) modulation of Akt phosphorylation via alpha7 nAChR and NMDA receptors. Neurobiol Aging.

[249] O'Reilly KE, Rojo F, She QB, Solit D, Mills GB, Smith D, Lane H, Hofmann F, Hicklin DJ, Ludwig DL (2006). mTOR inhibition induces upstream receptor tyrosine kinase signaling and activates Akt. Cancer Res.

[250] Sun SY, Rosenberg LM, Wang X, Zhou Z, Yue P, Fu H, Khuri FR (2005). Activation of Akt and eIF4E survival pathways by rapamycin-mediated mammalian target of rapamycin inhibition. Cancer Res.

[251] Bockaert J, Marin P (2015). mTOR in Brain Physiology and Pathologies. Physiol Rev.

[252] Lan AP, Chen J, Zhao Y, Chai Z, Hu Y (2017). mTOR Signaling in Parkinson's Disease. NeuroMolecular Med.

[253] Malagelada C, Ryu EJ, Biswas SC, Jackson-Lewis V, Greene LA (2006). RTP801 is elevated in Parkinson brain substantia nigral neurons and mediates death in cellular models of Parkinson's disease by a mechanism involving mammalian target of rapamycin inactivation. J Neurosci.

[254] Xu Y, Liu C, Chen S, Ye Y, Guo M, Ren Q, Liu L, Zhang H, Xu C, Zhou Q (2014). Activation of AMPK and inactivation of Akt result in suppression of mTOR-mediated S6K1 and 4E-BP1 pathways leading to neuronal cell death in in vitro models of Parkinson's disease. Cell Signal.

[255] Ciccone S, Maiani E, Bellusci G, Diederich M, Gonfloni S (2013). Parkinson's disease: a complex interplay of mitochondrial DNA alterations and oxidative stress. Int J Mol Sci.

[256] Perier C, Tieu K, Guegan C, Caspersen C, Jackson-Lewis V, Carelli V, Martinuzzi A, Hirano M, Przedborski S, Vila M (2005). Complex I deficiency primes Bax-dependent neuronal apoptosis through mitochondrial oxidative damage. Proc Natl Acad Sci U S A.

[257] Zhou Q, Liu C, Liu W, Zhang H, Zhang R, Liu J, Zhang J, Xu C, Liu L, Huang S, Chen L (2015). Rotenone induction of hydrogen peroxide inhibits mTOR-mediated S6K1 and 4E-BP1/eIF4E pathways, leading to neuronal apoptosis. Toxicol Sci.

[258] Chen L, Xu B, Liu L, Luo Y, Yin J, Zhou H, Chen W, Shen T, Han X, Huang S (2010). Hydrogen peroxide inhibits mTOR signaling by activation of AMPKalpha leading to apoptosis of neuronal cells. Lab Investig.

[259] Choi KC, Kim SH, Ha JY, Kim ST, Son JH (2010). A novel mTOR activating protein protects dopamine neurons against oxidative stress by repressing autophagy related cell death. J Neurochem.

[260] Corradetti MN, Inoki K, Guan KL (2005). The stress-inducted proteins RTP801 and RTP801L are negative regulators of the mammalian target of rapamycin pathway. J Biol Chem.

[261] Malagelada C, Jin ZH, Greene LA (2008). RTP801 is induced in Parkinson's disease and mediates neuron death by inhibiting Akt phosphorylation/activation. J Neurosci.

[262] Giacoppo S, Bramanti P, Mazzon E (2017). Triggering of inflammasome by impaired autophagy in response to acute experimental Parkinson's disease: involvement of the PI3K/Akt/mTOR pathway. Neuroreport.

[263] Malagelada C, Jin ZH, Jackson-Lewis V, Przedborski S, Greene LA (2010). Rapamycin protects against neuron death in in vitro and in vivo models of Parkinson's disease. J Neurosci.

[264] Ben Sahra I, Regazzetti C, Robert G, Laurent K, Le Marchand-Brustel Y, Auberger P, Tanti JF, Giorgetti-Peraldi S, Bost F (2011). Metformin, independent of AMPK, induces mTOR inhibition and cell-cycle arrest through REDD1. Cancer Res.

[265] Siracusa R, Paterniti I, Cordaro M, Crupi R, Bruschetta G, Campolo M, Cuzzocrea S, Esposito E (2018). Neuroprotective Effects of Temsirolimus in Animal Models of Parkinson's Disease. Mol Neurobiol.

[266] Masini D, Bonito-Oliva A, Bertho M, Fisone G (2018). Inhibition of mTORC1 Signaling Reverts Cognitive and Affective Deficits in a Mouse Model of Parkinson's Disease. Front Neurol.

[267] Ramalingam M, Huh YJ, Lee YI (2019). The Impairments of alpha-Synuclein and Mechanistic Target of Rapamycin in Rotenone-Induced SH-SY5Y Cells and Mice Model of Parkinson's Disease. Front Neurosci.

[268] Irwin DJ, Lee VM, Trojanowski JQ (2013). Parkinson's disease dementia: convergence of alpha-synuclein, tau and amyloid-beta pathologies. Nat Rev Neurosci.

[269] Crews L, Spencer B, Desplats P, Patrick C, Paulino A, Rockenstein E, Hansen L, Adame A, Galasko D, Masliah E (2010). Selective molecular alterations in the autophagy pathway in patients with Lewy body disease and in models of alpha-synucleinopathy. PLoS One.

[270] Menzies FM, Fleming A, Rubinsztein DC (2015). Compromised autophagy and neurodegenerative diseases. Nat Rev Neurosci.

[271] Scrivo A, Bourdenx M, Pampliega O, Cuervo AM (2018). Selective autophagy as a potential therapeutic target for neurodegenerative disorders. Lancet Neurol.

[272] Chu Y, Dodiya H, Aebischer P, Olanow CW, Kordower JH (2009). Alterations in lysosomal and proteasomal markers in Parkinson's disease: relationship to alpha-synuclein inclusions. Neurobiol Dis.

[273] Dijkstra AA, Ingrassia A, de Menezes RX, van Kesteren RE, Rozemuller AJ, Heutink P, van de Berg WD (2015). Evidence for Immune Response, Axonal Dysfunction and Reduced Endocytosis in the Substantia Nigra in Early Stage Parkinson's Disease. PLoS One.

[274] Gao S, Duan C, Gao G, Wang X, Yang H (2015). Alpha-synuclein overexpression negatively regulates insulin receptor substrate 1 by activating mTORC1/S6K1 signaling. Int J Biochem Cell Biol.

[275] Xiong R, Zhou W, Siegel D, Kitson RR, Freed CR, Moody CJ, Ross D (2015). A Novel Hsp90 Inhibitor Activates Compensatory Heat Shock Protein Responses and Autophagy and Alleviates Mutant A53T alpha-Synuclein Toxicity. Mol Pharmacol.

[276] Perez-Revuelta BI, Hettich MM, Ciociaro A, Rotermund C, Kahle PJ, Krauss S, Di Monte DA (2014). Metformin lowers Ser-129 phosphorylated alpha-synuclein levels via mTOR-dependent protein phosphatase 2A activation. Cell Death Dis.

[277] Hussain S, Feldman AL, Das C, Ziesmer SC, Ansell SM, Galardy PJ (2013). Ubiquitin hydrolase UCH-L1 destabilizes mTOR complex 1 by antagonizing DDB1-CUL4-mediated ubiquitination of raptor. Mol Cell Biol.

[278] Murata H, Sakaguchi M, Jin Y, Sakaguchi Y, Futami J, Yamada H, Kataoka K, Huh NH (2011). A new cytosolic pathway from a Parkinson disease-associated kinase, BRPK/PINK1: activation of AKT via mTORC2. J Biol Chem.

[279] Yarchoan M, Toledo JB, Lee EB, Arvanitakis Z, Kazi H, Han LY, Louneva N, Lee VM, Kim SF, Trojanowski JQ, Arnold SE (2014). Abnormal serine phosphorylation of insulin receptor substrate 1 is associated with tau pathology in Alzheimer's disease and tauopathies. Acta Neuropathol.

[280] Mahoney SJ, Narayan S, Molz L, Berstler LA, Kang SA, Vlasuk GP, Saiah E (2018). A small molecule inhibitor of Rheb selectively targets mTORC1 signaling. Nat Commun.

[281] Liu Y, Liu F, Grundke-Iqbal I, Iqbal K, Gong CX (2011). Deficient brain insulin signalling pathway in Alzheimer's disease and diabetes. J Pathol.

[282] Rickle A, Bogdanovic N, Volkman I, Winblad B, Ravid R, Cowburn RF (2004). Akt activity in Alzheimer's disease and other neurodegenerative disorders. Neuroreport.

[283] Chang RC, Wong AK, Ng HK, Hugon J (2002). Phosphorylation of eukaryotic initiation factor-2alpha (eIF2alpha) is associated with neuronal degeneration in Alzheimer's disease. Neuroreport.

[284] Wills J, Credle J, Oaks AW, Duka V, Lee JH, Jones J, Sidhu A (2012). Paraquat, but not maneb, induces synucleinopathy and tauopathy in striata of mice through inhibition of proteasomal and autophagic pathways. PLoS One.

[285] Martin-Flores N, Perez-Sisques L, Creus-Muncunill J, Masana M, Gines S, Alberch J, Perez-Navarro E, Malagelada C (2020). Synaptic RTP801 contributes to motor-learning dysfunction in Huntington's disease. Cell Death Dis.

[286] Creus-Muncunill J, Rue L, Alcala-Vida R, Badillos-Rodriguez R, Romani-Aumedes J, Marco S, Alberch J, Perez-Otano I, Malagelada C, Perez-Navarro E (2018). Increased Levels of Rictor Prevent Mutant Huntingtin-Induced Neuronal Degeneration. Mol Neurobiol.

[287] Creus-Muncunill J, Badillos-Rodriguez R, Garcia-Forn M, Masana M, Garcia-Diaz Barriga G, Guisado-Corcoll A, Alberch J, Malagelada C, Delgado-Garcia JM, Gruart A, Perez-Navarro E (2019). Increased translation as a novel pathogenic mechanism in Huntington's disease. Brain.

[288] Manzoni C, Mamais A, Roosen DA, Dihanich S, Soutar MP, Plun-Favreau H, Bandopadhyay R, Hardy J, Tooze SA, Cookson MR, Lewis PA (2016). mTOR independent regulation of macroautophagy by Leucine Rich Repeat Kinase 2 via Beclin-1. Sci Rep.

[289] Zhang Y, Nguyen DT, Olzomer EM, Poon GP, Cole NJ, Puvanendran A, Phillips BR, Hesselson D (2017). Rescue of Pink1 Deficiency by Stress-Dependent Activation of Autophagy. Cell Chem Biol.

[290] Jiang TF, Zhang YJ, Zhou HY, Wang HM, Tian LP, Liu J, Ding JQ, Chen SD (2013). Curcumin ameliorates the neurodegenerative pathology in A53T alpha-synuclein cell model of Parkinson's disease through the downregulation of mTOR/p70S6K signaling and the recovery of macroautophagy. J NeuroImmune Pharmacol.

[291] Redmann M, Wani WY, Volpicelli-Daley L, Darley-Usmar V, Zhang J (2017). Trehalose does not improve neuronal survival on exposure to alpha-synuclein pre-formed fibrils. Redox Biol.

[292] Sarkar S, Davies JE, Huang Z, Tunnacliffe A, Rubinsztein DC (2007). Trehalose, a novel mTOR-independent autophagy enhancer, accelerates the clearance of mutant huntingtin and alpha-synuclein. J Biol Chem.

[293] Castillo K, Nassif M, Valenzuela V, Rojas F, Matus S, Mercado G, Court FA, van Zundert B, Hetz C (2013). Trehalose delays the progression of amyotrophic lateral sclerosis by enhancing autophagy in motoneurons. Autophagy.

[294] Pupyshev AB, Tikhonova MA, Akopyan AA, Tenditnik MV, Dubrovina NI, Korolenko TA (2019). Therapeutic activation of autophagy by combined treatment with rapamycin and trehalose in a mouse MPTP-induced model of Parkinson's disease. Pharmacol Biochem Behav.

[295] Milnerwood AJ, Cummings DM, Dallerac GM, Brown JY, Vatsavayai SC, Hirst MC, Rezaie P, Murphy KP (2006). Early development of aberrant synaptic plasticity in a mouse model of Huntington's disease. Hum Mol Genet.

[296] Murphy KP, Carter RJ, Lione LA, Mangiarini L, Mahal A, Bates GP, Dunnett SB, Morton AJ (2000). Abnormal synaptic plasticity and impaired spatial cognition in mice transgenic for exon 1 of the human Huntington's disease mutation. J Neurosci.

[297] Usdin MT, Shelbourne PF, Myers RM, Madison DV (1999). Impaired synaptic plasticity in mice carrying the Huntington's disease mutation. Hum Mol Genet.

[298] Berger Z, Ravikumar B, Menzies FM, Oroz LG, Underwood BR, Pangalos MN, Schmitt I, Wullner U, Evert BO, O'Kane CJ, Rubinsztein DC (2006). Rapamycin alleviates toxicity of different aggregate-prone proteins. Hum Mol Genet.

[299] Roscic A, Baldo B, Crochemore C, Marcellin D, Paganetti P (2011). Induction of autophagy with catalytic mTOR inhibitors reduces huntingtin aggregates in a neuronal cell model. J Neurochem.

[300] Ravikumar B, Vacher C, Berger Z, Davies JE, Luo S, Oroz LG, Scaravilli F, Easton DF, Duden R, O'Kane CJ, Rubinsztein DC (2004). Inhibition of mTOR induces autophagy and reduces toxicity of polyglutamine expansions in fly and mouse models of Huntington disease. Nat Genet.

[301] Pryor WM, Biagioli M, Shahani N, Swarnkar S, Huang WC, Page DT, MacDonald ME, Subramaniam S (2014). Huntingtin promotes mTORC1 signaling in the pathogenesis of Huntington's disease. Sci Signal.

[302] Sarkar S, Ravikumar B, Floto RA, Rubinsztein DC (2009). Rapamycin and mTOR-independent autophagy inducers ameliorate toxicity of polyglutamine-expanded huntingtin and related proteinopathies. Cell Death Differ.

[303] Sarkar S, Krishna G, Imarisio S, Saiki S, O'Kane CJ, Rubinsztein DC (2008). A rational mechanism for combination treatment of Huntington's disease using lithium and rapamycin. Hum Mol Genet.

[304] Lee JH, Tecedor L, Chen YH, Monteys AM, Sowada MJ, Thompson LM, Davidson BL (2015). Reinstating aberrant mTORC1 activity in Huntington's disease mice improves disease phenotypes. Neuron.

[305] Gines S, Ivanova E, Seong IS, Saura CA, MacDonald ME (2003). Enhanced Akt signaling is an early pro-survival response that reflects N-methyl-D-aspartate receptor activation in Huntington's disease knock-in striatal cells. J Biol Chem.

[306] Saavedra A, Garcia-Martinez JM, Xifro X, Giralt A, Torres-Peraza JF, Canals JM, Diaz-Hernandez M, Lucas JJ, Alberch J, Perez-Navarro E (2010). PH domain leucine-rich repeat protein phosphatase 1 contributes to maintain the activation of the PI3K/Akt pro-survival pathway in Huntington's disease striatum. Cell Death Differ.

[307] Ji YJ, Ugolino J, Brady NR, Hamacher-Brady A, Wang J (2017). Systemic deregulation of autophagy upon loss of ALS- and FTD-linked C9orf72. Autophagy.

[308] Khayati K, Antikainen H, Bonder EM, Weber GF, Kruger WD, Jakubowski H, Dobrowolski R (2017). The amino acid metabolite homocysteine activates mTORC1 to inhibit autophagy and form abnormal proteins in human neurons and mice. FASEB J.

[309] Zhang X, Li L, Chen S, Yang D, Wang Y, Zhang X, Wang Z, Le W (2011). Rapamycin treatment augments motor neuron degeneration in SOD1(G93A) mouse model of amyotrophic lateral sclerosis. Autophagy.

[310] Hsueh KW, Chiou TW, Chiang SF, Yamashita T, Abe K, Borlongan CV, Sanberg PR, Huang AY, Lin SZ, Harn HJ (2016). Autophagic down-regulation in motor neurons remarkably prolongs the survival of ALS mice. Neuropharmacology.

[311] Saxena S, Roselli F, Singh K, Leptien K, Julien JP, Gros-Louis F, Caroni P (2013). Neuroprotection through excitability and mTOR required in ALS motoneurons to delay disease and extend survival. Neuron.

[312] Zhang X, Chen S, Song L, Tang Y, Shen Y, Jia L, Le W (2014). MTOR-independent, autophagic enhancer trehalose prolongs motor neuron survival and ameliorates the autophagic flux defect in a mouse model of amyotrophic lateral sclerosis. Autophagy.

[313] Holler CJ, Taylor G, McEachin ZT, Deng Q, Watkins WJ, Hudson K, Easley CA, Hu WT, Hales CM, Rossoll W (2016). Trehalose upregulates progranulin expression in human and mouse models of GRN haploinsufficiency: a novel therapeutic lead to treat frontotemporal dementia. Mol Neurodegener.

[314] Wang IF, Tsai KJ, Shen CK (2013). Autophagy activation ameliorates neuronal pathogenesis of FTLD-U mice: a new light for treatment of TARDBP/TDP-43 proteinopathies. Autophagy.

[315] Mandrioli J, D'Amico R, Zucchi E, Gessani A, Fini N, Fasano A, Caponnetto C, Chio A, Dalla Bella E, Lunetta C (2018). Rapamycin treatment for amyotrophic lateral sclerosis: Protocol for a phase II randomized, double-blind, placebo-controlled, multicenter, clinical trial (RAP-ALS trial). Medicine (Baltimore).

[316] Garber K (2009). Targeting mTOR: something old, something new. J Natl Cancer Inst.

[317] Sato A, Sunayama J, Matsuda K, Tachibana K, Sakurada K, Tomiyama A, Kayama T, Kitanaka C (2010). Regulation of neural stem/progenitor cell maintenance by PI3K and mTOR. Neurosci Lett.

[318] Li L (2017). The Molecular Mechanism of Glucagon-Like Peptide-1 Therapy in Alzheimer's Disease, Based on a Mechanistic Target of Rapamycin Pathway. CNS Drugs.

[319] McClean PL, Jalewa J, Holscher C (2015). Prophylactic liraglutide treatment prevents amyloid plaque deposition, chronic inflammation and memory impairment in APP/PS1 mice. Behav Brain Res.

[320] Maiese K (2016). Targeting molecules to medicine with mTOR, autophagy and neurodegenerative disorders. Br J Clin Pharmacol.

[321] Shi GD, OuYang YP, Shi JG, Liu Y, Yuan W, Jia LS (2011). PTEN deletion prevents ischemic brain injury by activating the mTOR signaling pathway. Biochem Biophys Res Commun.

[322] Chong ZZ, Shang YC, Wang S, Maiese K (2012). PRAS40 is an integral regulatory component of erythropoietin mTOR signaling and cytoprotection. PLoS One.

[323] Shang YC, Chong ZZ, Wang S, Maiese K (2012). Prevention of beta-amyloid degeneration of microglia by erythropoietin depends on Wnt1, the PI 3-K/mTOR pathway, Bad, and Bcl-xL. Aging (Albany NY).

[324] Cammalleri M, Lutjens R, Berton F, King AR, Simpson C, Francesconi W, Sanna PP (2003). Time-restricted role for dendritic activation of the mTOR-p70S6K pathway in the induction of late-phase long-term potentiation in the CA1. Proc Natl Acad Sci U S A.

[325] Vickers CA, Dickson KS, Wyllie DJ (2005). Induction and maintenance of late-phase long-term potentiation in isolated dendrites of rat hippocampal CA1 pyramidal neurones. J Physiol.

[326] Kelleher RJ, Govindarajan A, Tonegawa S (2004). Translational regulatory mechanisms in persistent forms of synaptic plasticity. Neuron.

[327] Graber TE, McCamphill PK, Sossin WS (2013). A recollection of mTOR signaling in learning and memory. Learn Mem.

[328] Maiese K (2014). Driving neural regeneration through the mammalian target of rapamycin. Neural Regen Res.

[329] Zhang L, Wang L, Wang R, Gao Y, Che H, Pan Y, Fu P (2017). Evaluating the Effectiveness of GTM-1, Rapamycin, and Carbamazepine on Autophagy and Alzheimer Disease. Med Sci Monit.

[330] Ehninger D, Han S, Shilyansky C, Zhou Y, Li W, Kwiatkowski DJ, Ramesh V, Silva AJ (2008). Reversal of learning deficits in a Tsc2+/- mouse model of tuberous sclerosis. Nat Med.

[331] Cai Z, Chen G, He W, Xiao M, Yan LJ (2015). Activation of mTOR: a culprit of Alzheimer's disease?. Neuropsychiatr Dis Treat.

[332] Zhao H, Wang ZC, Wang KF, Chen XY (2015). Abeta peptide secretion is reduced by Radix Polygalae-induced autophagy via activation of the AMPK/mTOR pathway. Mol Med Rep.

[333] Ramirez AE, Pacheco CR, Aguayo LG, Opazo CM (2014). Rapamycin protects against Abeta-induced synaptotoxicity by increasing presynaptic activity in hippocampal neurons. Biochim Biophys Acta.

[334] Spencer B, Potkar R, Trejo M, Rockenstein E, Patrick C, Gindi R, Adame A, Wyss-Coray T, Masliah E (2009). Beclinfkinson's and Lewy body diseases. J Neurosci.

[335] Van Skike CE, Jahrling JB, Olson AB, Sayre NL, Hussong SA, Ungvari Z, Lechleiter JD, Galvan V (2018). Inhibition of mTOR protects the blood-brain barrier in models of Alzheimer's disease and vascular cognitive impairment. Am J Physiol Heart Circ Physiol.

[336] Chen A, Xiong LJ, Tong Y, Mao M (2013). Neuroprotective effect of brain-derived neurotrophic factor mediated by autophagy through the PI3K/Akt/mTOR pathway. Mol Med Rep.

[337] Um SH, Frigerio F, Watanabe M, Picard F, Joaquin M, Sticker M, Fumagalli S, Allegrini PR, Kozma SC, Auwerx J, Thomas G (2004). Absence of S6K1 protects against age- and diet-induced obesity while enhancing insulin sensitivity. Nature.

[338] Li J, Kim SG, Blenis J (2014). Rapamycin: one drug, many effects. Cell Metab.

[339] Sadowski K, Kotulska K, Jozwiak S (2016). Management of side effects of mTOR inhibitors in tuberous sclerosis patients. Pharmacol Rep.

[340] Pereira MJ, Palming J, Rizell M, Aureliano M, Carvalho E, Svensson MK, Eriksson JW (2012). mTOR inhibition with rapamycin causes impaired insulin signalling and glucose uptake in human subcutaneous and omental adipocytes. Mol Cell Endocrinol.

[341] Yang SB, Lee HY, Young DM, Tien AC, Rowson-Baldwin A, Shu YY, Jan YN, Jan LY (2012). Rapamycin induces glucose intolerance in mice by reducing islet mass, insulin content, and insulin sensitivity. J Mol Med (Berl).

[342] Tyler B, Wadsworth S, Recinos V, Mehta V, Vellimana A, Li K, Rosenblatt J, Do H, Gallia GL, Siu IM (2011). Local delivery of rapamycin: a toxicity and efficacy study in an experimental malignant glioma model in rats. Neuro-Oncology.

[343] Dong X (2018). Current Strategies for Brain Drug Delivery. Theranostics.

[344] Kadakia E, Harpude P, Parayath N, Bottino D, Amiji M (2019). Challenging the CNS Targeting Potential of Systemically Administered Nanoemulsion Delivery Systems: a Case Study with Rapamycin-Containing Fish Oil Nanoemulsions in Mice. Pharm Res.

[345] Tramutola A, Lanzillotta C, Barone E, Arena A, Zuliani I, Mosca L, Blarzino C, Butterfield DA, Perluigi M, Di Domenico F (2018). Intranasal rapamycin ameliorates Alzheimer-like cognitive decline in a mouse model of Down syndrome. Transl Neurodegener.

[346] Floto RA, Sarkar S, Perlstein EO, Kampmann B, Schreiber SL, Rubinsztein DC (2007). Small molecule enhancers of rapamycin-induced TOR inhibition promote autophagy, reduce toxicity in Huntington's disease models and enhance killing of mycobacteria by macrophages. Autophagy.

[347] Benjamin D, Colombi M, Moroni C, Hall MN (2011). Rapamycin passes the torch: a new generation of mTOR inhibitors. Nat Rev Drug Discov.

[348] Chiarini F, Evangelisti C, McCubrey JA, Martelli AM (2015). Current treatment strategies for inhibiting mTOR in cancer. Trends Pharmacol Sci.

[349] Jiang T, Yu JT, Zhu XC, Tan MS, Wang HF, Cao L, Zhang QQ, Shi JQ, Gao L, Qin H (2014). Temsirolimus promotes autophagic clearance of amyloid-beta and provides protective effects in cellular and animal models of Alzheimer's disease. Pharmacol Res.

[350] Jiang T, Yu JT, Zhu XC, Zhang QQ, Cao L, Wang HF, Tan MS, Gao Q, Qin H, Zhang YD, Tan L (2014). Temsirolimus attenuates tauopathy in vitro and in vivo by targeting tau hyperphosphorylation and autophagic clearance. Neuropharmacology.

[351] Cassano T, Magini A, Giovagnoli S, Polchi A, Calcagnini S, Pace L, Lavecchia MA, Scuderi C, Bronzuoli MR, Ruggeri L (2019). Early intrathecal infusion of everolimus restores cognitive function and mood in a murine model of Alzheimer's disease. Exp Neurol.

[352] Imfeld P, Bodmer M, Jick SS, Meier CR (2012). Metformin, other antidiabetic drugs, and risk of Alzheimer's disease: a population-based case-control study. J Am Geriatr Soc.

[353] Koenig AM, Mechanic-Hamilton D, Xie SX, Combs MF, Cappola AR, Xie L, Detre JA, Wolk DA, Arnold SE (2017). Effects of the Insulin Sensitizer Metformin in Alzheimer Disease: Pilot Data From a Randomized Placebo-controlled Crossover Study. Alzheimer Dis Assoc Disord.

[354] Park SY, Lee HR, Lee WS, Shin HK, Kim HY, Hong KW, Kim CD (2016). Cilostazol Modulates Autophagic Degradation of beta-Amyloid Peptide via SIRT1-Coupled LKB1/AMPKalpha Signaling in Neuronal Cells. PLoS One.

[355] Salminen A, Kaarniranta K, Haapasalo A, Soininen H, Hiltunen M (2011). AMP-activated protein kinase: a potential player in Alzheimer's disease. J Neurochem.

[356] Wang C, Zhang X, Teng Z, Zhang T, Li Y (2014). Downregulation of PI3K/Akt/mTOR signaling pathway in curcumin-induced autophagy in APP/PS1 double transgenic mice. Eur J Pharmacol.

[357] Sarkar S, Perlstein EO, Imarisio S, Pineau S, Cordenier A, Maglathlin RL, Webster JA, Lewis TA, O'Kane CJ, Schreiber SL, Rubinsztein DC (2007). Small molecules enhance autophagy and reduce toxicity in Huntington's disease models. Nat Chem Biol.

[358] Vieira MNN, Lima-Filho RAS, De Felice FG (2018). Connecting Alzheimer's disease to diabetes: Underlying mechanisms and potential therapeutic targets. Neuropharmacology.

[359] Butterfield DA, Boyd-Kimball D (2018). Oxidative Stress, Amyloid-beta Peptide, and Altered Key Molecular Pathways in the Pathogenesis and Progression of Alzheimer's Disease. J Alzheimers Dis.

[360] Liang H, Nie J, Van Skike CE, Valentine JM, Orr ME (2019). Mammalian Target of Rapamycin at the Crossroad Between Alzheimer's Disease and Diabetes. Adv Exp Med Biol.

[361] Mannaa M, Kramer S, Boschmann M, Gollasch M (2013). mTOR and regulation of energy homeostasis in humans. J Mol Med (Berl).

[362] Tischmeyer W, Schicknick H, Kraus M, Seidenbecher CI, Staak S, Scheich H, Gundelfinger ED (2003). Rapamycin-sensitive signalling in long-term consolidation of auditory cortex-dependent memory. Eur J Neurosci.

[363] Yang H, Shi O, Jin Y, Henrich-Noack P, Qiao H, Cai C, Tao H, Tian X (2014). Functional protection of learning and memory abilities in rats with vascular dementia. Restor Neurol Neurosci.

[364] Romine J, Gao X, Xu XM, So KF, Chen J (2015). The proliferation of amplifying neural progenitor cells is impaired in the aging brain and restored by the mTOR pathway activation. Neurobiol Aging.

[365] Xie R, Wang P, Ji X, Zhao H (2013). Ischemic post-conditioning facilitates brain recovery after stroke by promoting Akt/mTOR activity in nude rats. J Neurochem.

[366] Maiese K (2015). Neuronal Activity, Mitogens, and mTOR: Overcoming the Hurdles for the Treatment of Glioblastoma Multiforme. J Transl Sci.

[367] Chi OZ, Mellender SJ, Barsoum S, Liu X, Damito S, Weiss HR (2016). Effects of rapamycin pretreatment on blood-brain barrier disruption in cerebral ischemia-reperfusion. Neurosci Lett.

[368] Zare Mehrjerdi F, Aboutaleb N, Habibey R, Ajami M, Soleimani M, Arabian M, Niknazar S, Hossein Davoodi S, Pazoki-Toroudi H (2013). Increased phosphorylation of mTOR is involved in remote ischemic preconditioning of hippocampus in mice. Brain Res.

[369] Thoreen CC, Sabatini DM (2009). Rapamycin inhibits mTORC1, but not completely. Autophagy.

[370] Choo AY, Yoon SO, Kim SG, Roux PP, Blenis J (2008). Rapamycin differentially inhibits S6Ks and 4E-BP1 to mediate cell-type-specific repression of mRNA translation. Proc Natl Acad Sci U S A.

[371] Jahrling JB, Laberge RM (2015). Age-Related Neurodegeneration Prevention Through mTOR Inhibition: Potential Mechanisms and Remaining Questions. Curr Top Med Chem.

[372] Kaeberlein M, Galvan V (2019). Rapamycin and Alzheimer's disease: Time for a clinical trial?. Sci Transl Med.

